# Mapping of the *bs5* and *bs6* non-race-specific recessive resistances against bacterial spot of pepper

**DOI:** 10.3389/fpls.2023.1061803

**Published:** 2023-05-19

**Authors:** Anuj Sharma, Jian Li, Rebecca Wente, Gerald V. Minsavage, Upinder S. Gill, Arturo Ortega, C. Eduardo Vallejos, John P. Hart, Brian J. Staskawicz, Michael R. Mazourek, Robert E. Stall, Jeffrey B. Jones, Samuel F. Hutton

**Affiliations:** ^1^ Department of Plant Pathology, University of Florida, Gainesville, FL, United States; ^2^ Gulf Coast Research and Education Center, University of Florida, Wimauma, FL, United States; ^3^ Horticultural Sciences Department, University of Florida, Gainesville, FL, United States; ^4^ Department of Plant and Microbial Biology, University of California, Berkeley, Berkeley, CA, United States; ^5^ Innovative Genomics Institute, University of California, Berkeley, Berkeley, CA, United States; ^6^ Plant Breeding and Genetics Section, School of Integrative Plant Science, Cornell University, Ithaca, NY, United States

**Keywords:** *capsicum annuum*, genotyping-by-sequencing, *xanthomonas euvesicatoria*, disease resistance, marker-assisted selection, recessive resistance

## Abstract

Bacterial spot caused by *Xanthomonas euvesicatoria* is a major disease of pepper (*Capsicum annuum* L.) in warm and humid production environments. Use of genetically resistant cultivars is an effective approach to manage bacterial spot. Two recessive resistance genes, *bs5* and *bs6*, confer non-race-specific resistance against bacterial spot. The objective of our study was to map these two loci in the pepper genome. We used a genotyping-by-sequencing approach to initially map the position of the two resistances. Segregating populations for *bs5* and *bs6* were developed by crossing susceptible Early CalWonder (ECW) with near-isogenic lines ECW50R (*bs5* introgression) or ECW60R (*bs6* introgression). Following fine-mapping, *bs5* was delimited to a ~535 Kbp interval on chromosome 3, and *bs6* to a ~666 Kbp interval in chromosome 6. We identified 14 and 8 candidate resistance genes for *bs5* and *bs6*, respectively, based on predicted protein coding polymorphisms between ECW and the corresponding resistant parent. This research enhances marker-assisted selection of *bs5* and *bs6* in breeding programs and is a crucial step towards elucidating the molecular mechanisms underlying the resistances.

## Introduction

Pepper (*Capsicum annuum* L.) is an important solanaceous crop that is cultivated throughout the world. Bacterial spot of pepper (BSP) is a major disease responsible for loss of marketable yield in many pepper-growing regions (Osdaghi et al., 2021). The disease is manifested as dark brown necrotic lesions in all aerial parts of the plant. Foliar infection can lead to defoliation, which in turn leads to yield loss. The marketability of fresh fruits is also affected by the presence of scab-like symptoms or due to sun-scalding resulting from extensive defoliation (Ritchie, 2000). The disease is caused by three species of *Xanthomonas* — *X. vesicatoria*, *X. euvesicatoria* (*Xe*), and *X. gardneri* (*Xg*) (Osdaghi et al., 2021). The management of BSP often relies on application of copper-based bactericides; however, the emergence of copper-tolerant strains has rendered this strategy unsustainable (Stall et al., 2009). Alternatively, host plant resistance has been deployed as an effective, economical, and environmentally friendly way of mitigating economic damage caused by BSP.

Most of the resistances deployed in modern agriculture are conditioned by dominant resistance (R) genes which often belong to Nucleotide-Binding Leucine Rich Repeats (NLR) or Receptor-Like Kinase (RLK) protein families (Sharma et al., 2022a). Five dominant resistances have been reported against BSP — *Bs1* from *C. annuum* accession PI 163192 (Cook and Stall, 1963), *Bs2* from *C. chacoense* PI 260435 (Cook and Guevara, 1984), *Bs3* from *C. annuum* PI 271322 (Kim and Hartmann, 1985), *Bs4C* from *C. pubescens* PI 235047 (Sahin and Miller, 1998), and *Bs7* from *C. baccatum* var. *pendulum* UENF 1556 (Potnis et al., 2011). Among them, only *Bs2* and *Bs3*, and to some extent *Bs1*, have been commercially deployed. Based on gene-for-gene interactions between R genes and their corresponding avirulence genes, BSP causing *Xe* has been classified into eleven races (P0 – P10) (Stall et al., 2009). *Bs1* provides resistance against races P0, P2, and P5; *Bs2* against races P0, P1, P2, P3, P7, and P8; and *Bs3* against races P0, P1, P4, P7, and P9. Dominant resistance following infection often results in elicitation of a hypersensitive response (HR) and programmed cell death which creates high selection pressure for emergence and enrichment of pathogen races that overcome such resistance through loss/modification of avirulence genes (Gassmann et al., 2000). As a result, R genes are usually short-lived as exemplified by emergence and increased prevalence of races P6 and P10 in bell pepper cultivation, which are insensitive to the deployed R-genes (Kousik and Ritchie, 1996a; Kousik and Ritchie, 1996b; Kousik and Ritchie, 1998; Pernezny et al., 1999; Stall et al., 2009).

In contrast to R genes, recessive resistances typically result from the loss or modification of host susceptibility (S) factors that are exploited by bacteria to initiate a disease response (Sharma et al., 2022a). Recessive resistances are not race-specific and, following infection, do not elicit an HR — the lower selection pressure reduces the chance of emergence of resistance-breaking virulent strains (Parlevliet, 2002; Poland et al., 2009). This makes recessive resistance, despite the breeding challenges, highly desirable for management of rapidly evolving bacterial pathogens, such as *Xe*. Currently, three recessive resistances have been identified against BSP — *bs5* derived from *C. annuum* PI 271322, *bs6* from *C. annuum* PI 163192 or PI 264281, and *bs8* from *C. annuum* PI 163192 (Jones et al., 2002; Sharma et al., 2022b). Two of these genes, *bs5* and *bs6*, confer resistance to all known *Xe* races, including race P6 and P10 (Jones et al., 2002; Vallejos et al., 2010). Although *bs8* has been demonstrated to suppress *Xg*, its effect on *Xe* is not known (Sharma et al., 2022b). Only *bs5* has been commercially deployed (McCarthy, 2011; McCarthy, 2012), and there have been no reports of its suppression by *Xe*.

Both *bs5* and *bs6* were first reported as monogenic, recessive, non-HR resistances against *Xe* race P6 (Jones et al., 2002). Both resistance genes were derived from hot pepper accessions collected from India and maintained at the USDA Plant Genetic Resources Conservation Unit, GA (npgsweb.ars-grin.gov/gringlobal). *bs5* was reported to originate from *C. annuum* PI 271322 (Russell, 1955), which had previously been reported to carry field resistance against BSP (Sowell and Dempsey, 1977). Although *bs6* is described as originating from either PI 163192 or PI 264281, the most probable source is PI 163192 (Hyland, 1967), which Dempsey et al. (1981) utilized to incorporate bacterial spot resistance into the C44 series of pepper breeding lines; included in this series is the Pep13 line which was used as *bs6* donor by Jones et al. (2002) (Lane et al., 1997). Jones et al. (2002) transferred *bs5* to the bell pepper *C. annuum* Early CalWonder (ECW) background by repeated backcrosses to ultimately generate ECW-50R line (Vallejos et al., 2010). A similar strategy was used to develop an ECW NIL containing *bs6*, which has been named ECW-60R. Recent literature has uncovered that *bs5* is also present in PI 163192 (Szarka et al., 2022).

In order to understand the mechanism of resistance, it is often necessary to identify the underlying resistance gene. This is accomplished by gene mapping, which is the process of determining the physical location of a gene in the genome. Mapping of a resistance gene locus also enables the development and use of linked molecular markers (in addition to phenotypic selection) to accelerate the breeding process through marker-assisted selection. Genotyping-by-sequencing (GBS) is a robust sequencing-based method of surveying genome-wide polymorphisms which can be utilized to discover molecular markers (such as SNPs and InDels) and genotypes the samples with those markers in a single step (Elshire et al., 2011). As a large number of small genomic variations from all chromosomes can be utilized in mapping, GBS often provides higher resolution than traditional genotyping methods. In this paper, we (i) identified the genomic localization of *bs5* and *bs6* resistance genes in pepper genome using GBS, (ii) fine mapped the respective resistance regions and identified flanking markers, and (iii) identified and analyzed candidate resistance genes.

## Results

### Segregation and phenotype

**Figure 1:**
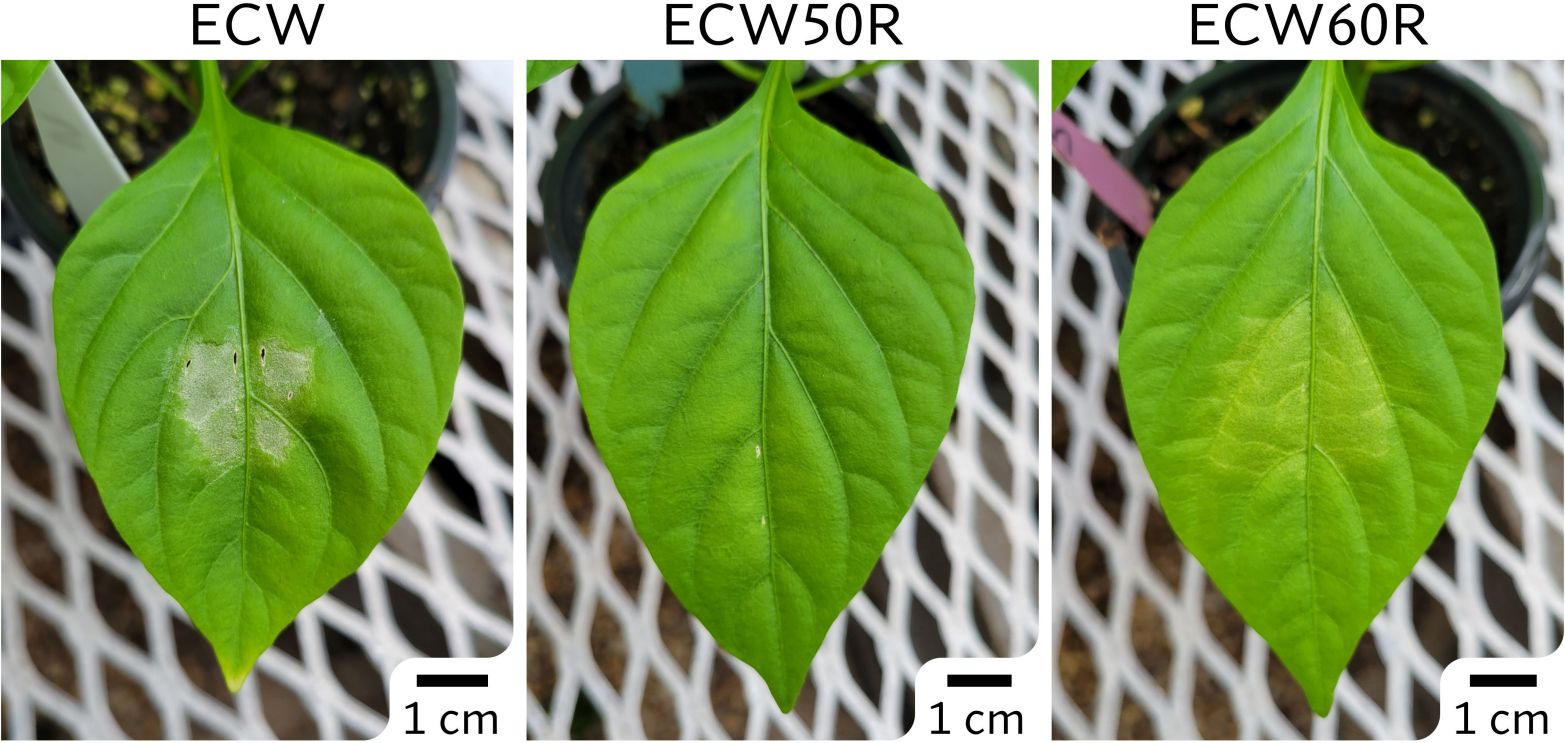
Phenotypes of ECW, ECW50R (*bs5*), and ECW60R (*bs6*) pepper 5 days after inoculation of *Xanthomonas euvesicatoria* strain Xv157 at 10^5^ CFU/ml.

The phenotypic differences between ECW and ECW50R (*bs5*) were clear and easily distinguishable following inoculation at a relatively low bacterial concentration (10^5^ CFU/ml) (**Figure 1**). The ECW leaf tissue developed necrotic lesions surrounded by yellow halos while the ECW50R tissue remained mostly green. In the GBS F_2_ population, 91 out of 100 F_2_s (19 resistant and 72 susceptible) were phenotyped with high confidence and thus were used for GBS step. The ratio of resistant to susceptible F_2_s (1:3.8) was slightly lower than the expected ratio of 1:3 for recessive monogenic inheritance, however the difference was not statistically significant (*X^2 =^ 0.824* at 1 degree of freedom; *p=0.364*).

The phenotype of ECW60R (*bs6*) resistance was not as distinct as *bs5* (**Figure 1**). As expected, *bs6* resistance was characterized by extensive chlorosis. Out of 120 F_2_s, 92 most clearly phenotyped individuals (29 resistant and 63 susceptible) were selected for GBS analysis. The ratio of resistant to susceptible F_2_s (1:2.2) was not statistically different (*X^2 =^ 2.087* at 1 degree of freedom; *p=0.1486*) from the expected 1:3 ratio.

### *bs5* locus is linked to shorter arm of chromosome 3

**Figure 2:**
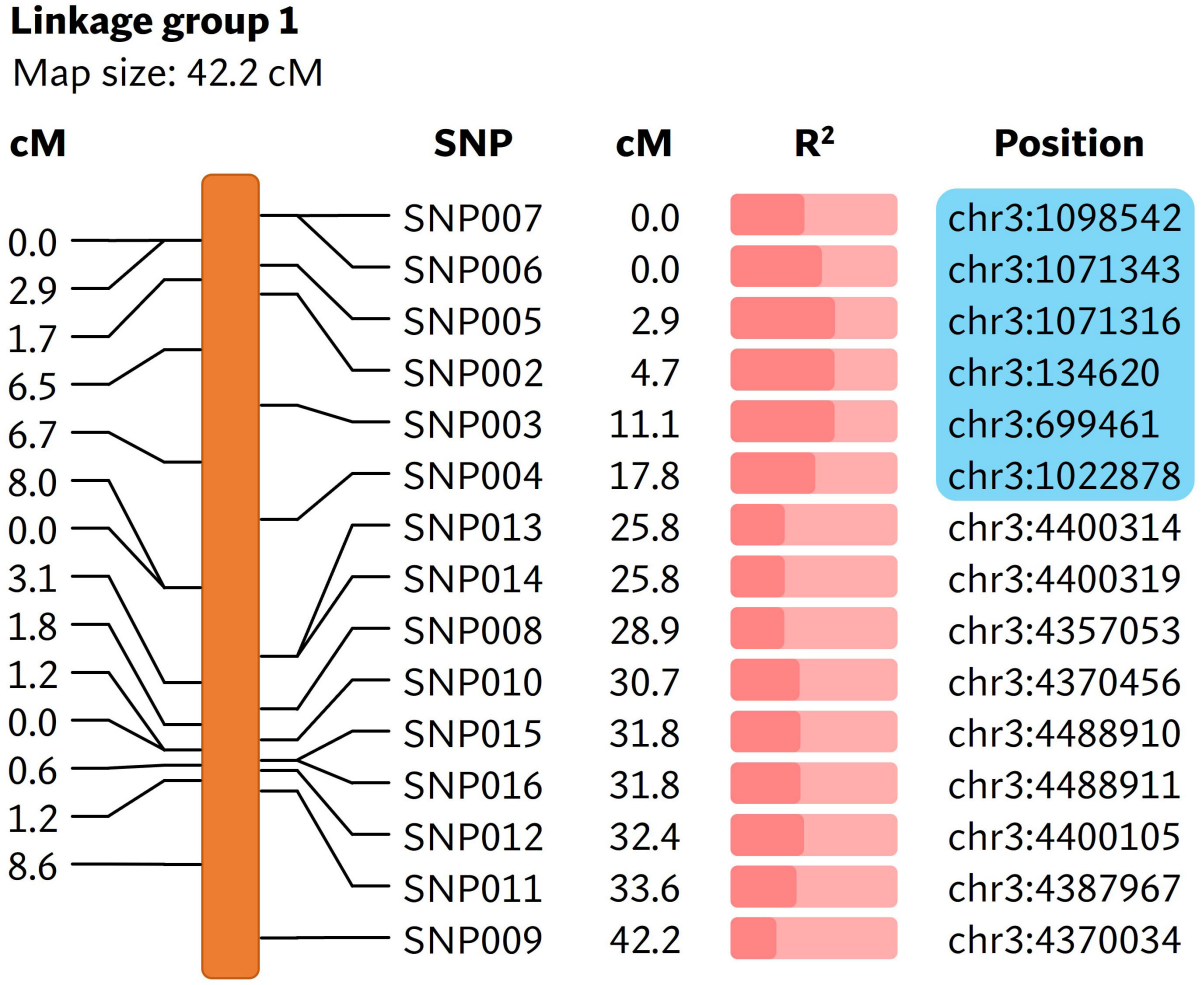
Linkage map showing markers associated with *bs5*. The cM values in the left represent the linkage distance between the markers and the cM values in the middle represent the positions of markers in the linakge group. The R^2^ value (**Supplementary Table 3**) is represented by the fraction of light salmon background filled by darker color. The physical positions of markers are based on *C. annuum* UCD10X genome, release 1.1. Blue box encloses genomic area that was further investigated by fine-mapping. cM, centimorgan; R^2^, coefficient of determination.

A total of 169,398,995 reads were generated from the *bs5* GBS library (**Supplementary Table 1**). The GBS pipeline discovered 101 high quality SNPs that were polymorphic between the two parents, and those SNPs were selected for further analysis. The linkage analysis of 88 F_2_s that could be genotyped identified thirteen linkage groups, and the *bs5* resistance mapped to linkage group 1 in chromosome 3 with highest significance (**Figure 2**; **Supplementary Tables 2**, **3**). SNPs between positions 134,620 and 1,098,542 of chromosome 3 were the most significantly associated with *bs5* (p<0.0001). Genotyping of the F_2_ population with CAPS markers spanning the linkage region confirmed 100% marker-trait co-segregation in the mapping population (**Supplementary Tables 4**, **5**). The results indicate that *bs5* is located towards the distal end of the short arm of chromosome 3, within a ~1 Mbp interval between 0.1 and 1.1 Mbp position.

### *bs5* is fine-mapped to a 546 Kbp interval in sub-telomeric region of chromosome 3

**Figure 3:**
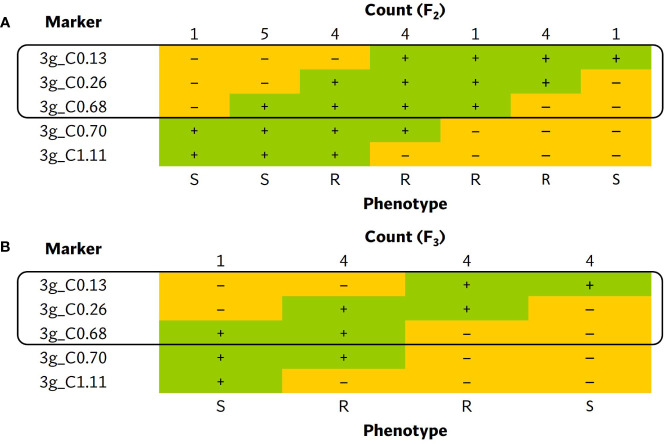
Tabulation of genotypes of the **(A)** F_2_ and **(B)** F_3_ progenies from *bs5* fine-mapping population that recombine within the *bs5* mapped region, together with their phenotypes. The black boxes enclose the closest markers flanking the new resistance interval. The numeric portion of marker names following “C” represent their approximate position (in megabases) in chromosome 3 of *C. annuum* UCD10X genome, release 1.1. +, homozygous for the resistant/ECW50R allele; –, heterozygous or homozygous for the susceptible/ECW allele; R, resistant phenotype; S, susceptible phenotype.

A larger ECW × ECW50R F_2_ population was developed to fine-map the position of *bs5*. Out of 1270 F_2_s genotyped with flanking markers 3g_C0.134 and 3g_C1.11 (**Supplementary Table 4**), 16 individuals were identified as recombinants and were phenotyped. Ten informative recombinants and F_3_ RILs developed from six non-informative recombinants placed *bs5* into an ~546 Kb interval between markers 3g_C0.134 (~0.4 cM) and 3g_C0.68 (~0.95 cM) with tight linkage with marker 3g_C0.26. (**Figure 3**; **Supplementary Tables 6**, **7**).

### *bs5* interval contains 14 polymorphic candidate genes

An ECW *bs5* super-scaffold was developed by concatenating *C. annuum* ECW scaffolds that align with in *C. annuum* UCD10X *bs5* interval. This super-scaffold consisted of 535 Kbp sequence including gaps and flanking region and provided complete coverage of UCD10X *bs5* interval (**Supplementary Table 8**). Comparison of whole genome polymorphisms between *bs5*-fixed line (PI 163192 × ECW50R) and ECW identified a total of 1,718 variants in this region under stringent filtration (data not shown). However, only 28 variants were found to alter the protein sequences, which resulted in 14 putative candidate genes for *bs5* resistance (**Table 1**; **Alignment S1-S14**).

**Table 1:** List of candidate genes for *bs5* resistance.

POS	REF	ALT	MUTATION	NUCL	PROT	GENEID	ANNOTATION
14617	A	G	missense	755T>C	252I>T	107864408	CMP-sialic acid transporter 2
15751	T	C	missense	481A>G	161M>V		
128730	A	C	missense	770T>G	257L>R	107864414	diacylglycerol lipase-β
150704	T	TCTCC ATTTC CAT	conservative inframe insertion	297+AT GGAAA TGGAG	101E>I +MEME	107864416	CRIB domain-containing protein
157383	A	C	missense	951T>G	317D>E	107864417	LRR protein kinase MSP1-like
158161	T	C	missense	173A>G	58H>R		
158243	C	G	missense	91G>C	31E>Q		
158320	C	A	missense	14G>T	5C>F		
158840	T	A	missense	2038A>T	680R>W	107866541	ABC transporter
169538	G	T	missense	238C>A	80Q>K		
175028	A	T	missense	203A>T	68K>M	107866543	glycine-rich protein
175034	A	G	missense	209A>G	70Y>C		
199255	C	G	missense	778G>C	260A>P	107864418	vacuolar AA transporter 1
223076	G	A	missense	398C>T	133P>T	107864422	ribosome biogenesis protein
223754	G	A	missense	371C>T	124T>I		
266460	A	AA	frameshift	1417+A	473S>fs	107864424	WD repeat-containing
270358	CCAA GAG	C	conservative inframe deletion	259–lC TCTTG	87–LL	107864425	cysteine-rich transmembrane domain protein
447967	G	A	missense	1912G>A	638A>T	107864431	ATP-dependent DNA helicase 2 subunit KU70
448066	G	A	missense	2011G>A	671G>S		
448486	TN_28_	T	frameshift	2072–N_28_	69S>fs		
466069	T	C	missense	2308A>G	770T>A	107864438	putative late blight resistance protein R1B-16
466599	G	A	missense	1778C>T	593A>V		
467671	T	C	missense	706A>G	236M>V		
467823	T	G	missense	554A>C	185K>T		
468340	C	G	missense	37G>C	13G>R		
523915	T	A	missense	168A>T	56E>D	107864444	
526217	G	A	missense	149C>T	50P>L		
528677	A	G	missense	151A>G	51I>V	107865674	pirin-like protein

REF, ECW/susceptible allele; ALT, ECW50R/resistant allele; NUCL, nucleotide change; PROT, amino acid change. The horizontal lines delineate different genes. ‘POS’ indicated position of polymorphism in bs5 super-scaffold (**Supplementary Table 8**). ‘GENEID’ is based on homology search with pepper reference genome in NCBI and the gene sequences used for variant annotation may vary from the sequences of genes listed in this column.

### *bs6* locus is located in chromosome 6

**Figure 4:**
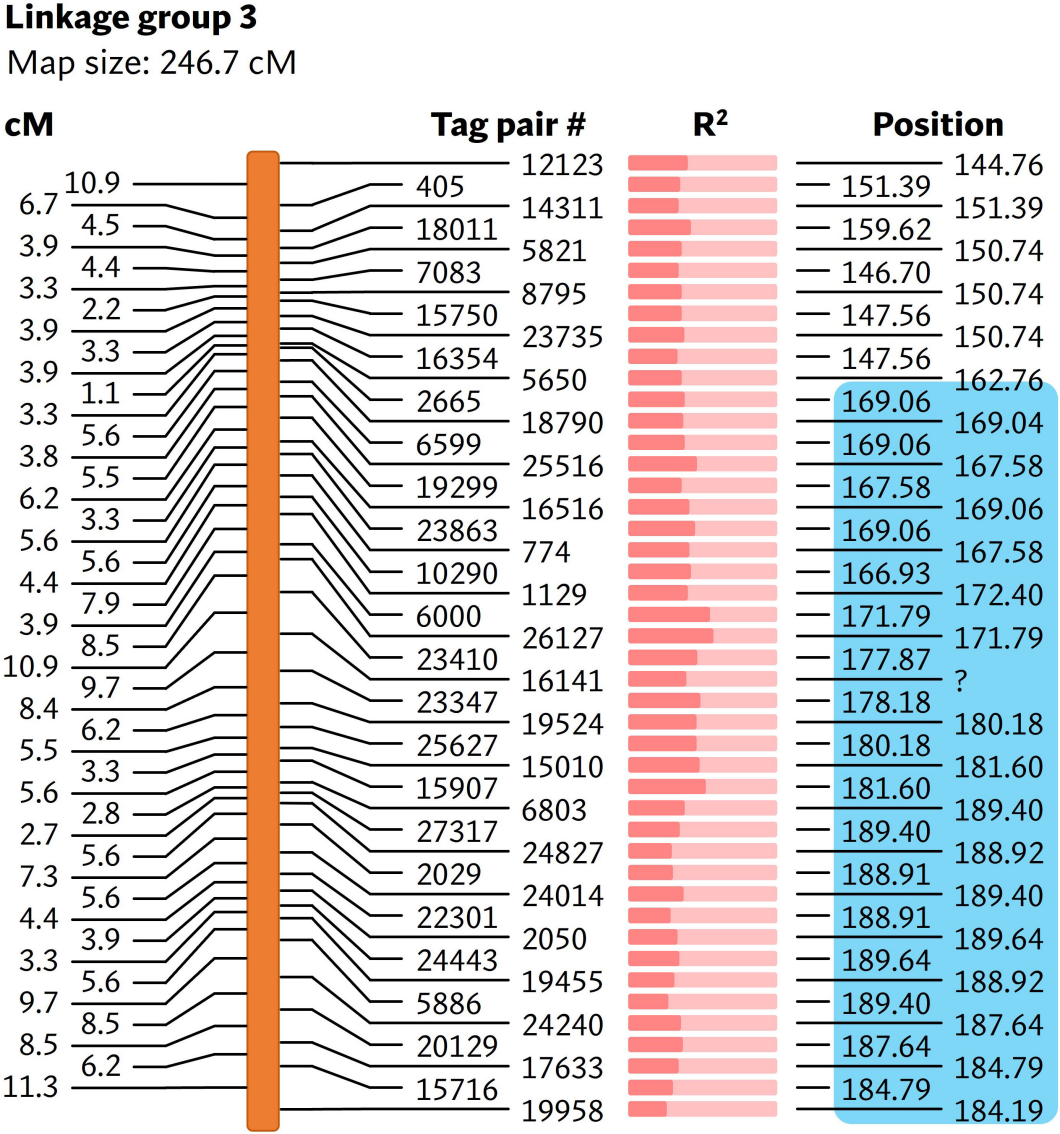
Linkage map showing markers associated with *bs6*. The cM values on the left represent the linkage distance between the markers. The R^2^ value (**Supplementary Table 11**) is represented by the fraction of light salmon background filled by darker color. The physical positions of the markers are based on chromosome 6 of *C. annuum* UCD10X genome, release 1.1. Blue box encloses genomic area that was further investigated by fine-mapping. cM, centimorgan; R^2^, coefficient of determination. #, number; ?, unmapped / unknown position.

As the reference-based GBS pipeline only identified a small number of polymorphic markers, the reference-free UNEAK pipeline was used for mapping *bs6*. This pipeline discovered 133 SNPs from a total of 173,074,228 reads generated from sequencing (**Supplementary Table 9**). Nine linkage groups were generated from the linkage analysis using genotyping information from 92 F_2_ plants (**Supplementary Table 10**), out of which the *bs6* resistance phenotype was significantly (p < 0.0001) linked to SNPs on linkage group 3 (**Figure 4**; **Supplementary Tables 10**, **11**). The linkage group was determined to be physically located in chromosome 6. CAPS markers were developed in the *bs6*-mapped region, and genotyping of the F_2_ population validated the linkage between those markers and the resistance phenotype (**Supplementary Tables 12**, **13**). The results indicated that *bs6* was located within an ~21 Mbp interval between positions 168–189 Mbp in *C. annuum* UCD10X genome.

### *bs6* is fine-mapped to a 656 Kb interval

**Figure 5:**
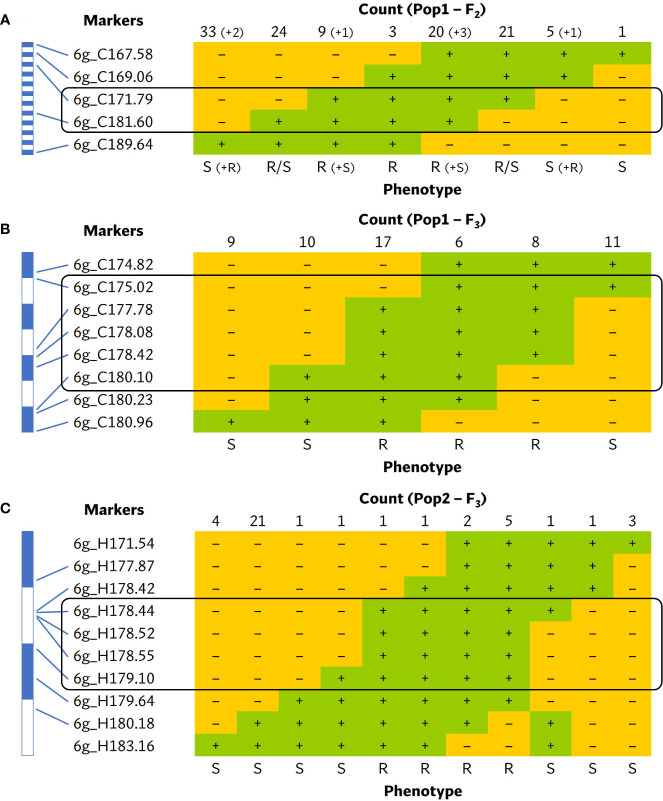
Tabulation of genotypes of the **(A)** F_2_ plants from first *bs6* fine-mapping population, **(B)** F_3_ plants from first *bs6* fine-mapping population, and **(C)** F_3_ plants from second *bs6* fine-mapping population that recombine within the *bs6* interval, together with their phenotypes. The black boxes enclose the closest markers flanking the new resistance interval. Each blue and white block in the scale bar on the left represents 1 Mbp region. The numeric portion of marker names following “C” or “H” represent their approximate position (in megabases) in chromosome 6 of *C. annuum* UCD10X genome, release 1.1. +, homozygous for the resistant/ECW60R allele; –, heterozygous or homozygous for the susceptible/ECW allele; R, resistant phenotype; S, susceptible phenotype.

Five of the CAPS markers within the ~21 Mbp *bs6* interval were initially used to more precisely determine the position of *bs6*. In a fine mapping F_2_ population of 940 plants, 277 plants were identified as recombinants, 123 of which were homozygous for 60R alleles throughout part of the recombined region and were phenotyped as F_2_ plants; genotyping of these F_2_s delimited the resistance locus to an ~9.8 Mbp region between markers 6g_C171.79 and 6g_C181.60 (**Figure 5A**; **Supplementary Table 14**). F_3_ RILs developed from 61 F_2_s that recombined within the region were genotyped with eight new CAPS markers within the interval (**Supplementary Table 12**); this delimited *bs6* within an ~5.1 Mbp interval between markers 6g_C175.02 and 6g_C180.10 (**Figure 5B**; **Supplementary Table 15**). A second ECW60R × ECW F_2_ population of 940 plants was developed and genotyped with new HRM markers (**Supplementary Table 12**), and 41 recombinants between flanking markers 6g_H171.54 and 6g_H183.16 were identified and developed into F3 RILs. All 41 RILs were phenotyped and were genotyped with markers in the 5.1 Kbp interval, thereby delimiting *bs6* to an ~656 Kbp region between markers 6g_H178.44 (~0.11 cM) and 6g_H179.10 (~0.11 cM) (**Figure 5C**; **Supplementary Table 16**).

### *bs6* interval contains 8 polymorphic candidate genes

The ECW *bs6* super-scaffold spanned three *C. annuum* ECW scaffolds with a total size of 681 Kb, providing complete coverage of UCD10X *bs6* interval (**Supplementary Table 8**). A total of 1,718 variants were identified between ECW and ECW60R genome in this region after filtration. Annotation of those variants identified protein coding changes in eight genes, which are candidates for *bs6* (**Table 2**; **Alignment S15-S22**). Interestingly, four of those candidates are functionally annotated as ZED1-related serine/threonine kinases, and three have protein polymorphisms within the putative kinase domain (**Table 2**).

**Table 2:** List of candidate genes for *bs6* resistance.

POS	REF	ALT	MUTATION	NUCL	PROT	GENEID	ANNOTATION
4324	G	A	stop gained	439C>T	147Q>*	107874896	Formyltetrahydrofolate deformylase
24554	G	A	missense	211C>T	71L>F	107872943	Phosphatidylserine decarboxylase proenzyme 1
49875	C	A	missense	255C>A	85D>E	107872942	ZED1-related kinase (ZRK) 4
49930	A	AT	frameshift	311+T	105A>fs		
50091	T	A	missense	471T>A	157N>K		
52304	T	TA	frameshift	111+A	38E>fs	107874893	ZRK1-like serine/ threonine-protein kinase
52358	C	T	missense	164C>T	55S>F		
52928	T	G	missense	457T>G	153S>A		
59302	C	T	missense	168G>A	56M>I	FXO38_32052	TCP-1/Cpn-60 chaperonin-like
59330	G	A	missense	140C>T	47S>F		
92420	A	T	missense	782A>T	261E>V	107874060	ZRK1-like serine/ threonine-protein kinase
92548	C	G	missense	910C>G	304P>A		
92596	C	A	missense	958C>A	320P>T		
94020	G	A	missense	122G>A	41G>D	FXO37_21555	Ubiquitin conjugating enzyme variant (UEV) 1C-like
94033	C	G	missense	135C>G	45I>M		
94050	C	G	missense	152C>G	51T>S		
94053	A	G	missense	155A>G	52D>G		
127705	TTAA	T	disruptive inframe deletion	308–ATA	103–N	107874895	ZED1-related kinase (ZRK) 1
128076	A	G	missense	673A>G	225K>E		

REF, ECW/susceptible allele; ALT, ECW60R/resistant allele; NUCL, nucleotide change; PROT, amino acid change. The horizontal lines delineate different genes. ‘POS’ indicated position of polymorphism in bs6 super-scaffold (**Supplementary Table 8**). ‘GENEID’ is based on homology search with pepper reference genome in NCBI and FXONN_NNNNN are proteins annotated in *C. annuum* ECW genome assembly. The gene sequences used for variant annotation may vary from the sequences of genes listed in this column.*, stop codon (standard AA symbol). fs, frameshift (standard notation).

## Discussion

In this paper, we determined the genomic localization of two recessive BSP resistance genes: *bs5* and *bs6*. *bs5* was mapped to the telomeric region of chromosome 3 and *bs6* to chromosome 6. The genomic position of *bs5* is in discordance with a previous report on the position of *bs5*, which had mapped it to the centromeric region of chromosome 6 (Vallejos et al., 2010). However, the chromosomal position in the previous study was based upon two populations of 60 F_2_ and 88 F_3_ progenies and only utilized 64 markers for screening the entire pepper genome. In contrast, the *bs5* locations identified in the present study benefited from a much larger number of markers identified through GBS, and has been validated in large fine mapping populations. Furthermore, the recent availability of a high-quality pepper reference genome enabled us to cross-validate our mapping results with the physical positions in the pepper chromosomes.

Several pepper lines have been reported to have varying degrees of recessive resistance against BSP. One of the earliest discoveries of recessive resistance was made by Dempsey (1953) in the pepper cultivar, Santanka. Hibberd et al. (1988) reported quantitative non-race-specific resistance in PI 163189. Poulos et al. (1992) reported that the quantitative, non-HR, non-race-specific resistance in CNPH 703 is controlled by at least two genes. Both PI 163189 and PI 183441 (parent of CNPH 703) were imported together with PI 163192, and thus the resistances in those accessions could also be due to *bs5*/*bs6*. A monogenic, recessive, non-HR and non-race-specific resistance in PI 163192 was identified by Szarka and Csilléry (2001) and named *gds* (general defense system); *gds* has since been shown to be the same as *bs5* (Timár et al., 2019). Riva et al. (2004) reported recessive resistance in UENF 1381 that may be governed by multiple genes. Furthermore, several genes have been identified in pepper which are required for complete virulence; reduced expression of such genes resulted in reduced susceptibility to BSP. Some notable examples include GLIP1 (Hong et al., 2008), MRP1 (An et al., 2008), MLO2 (Kim and Hwang, 2012), and GRP1 (Kim et al., 2015).

A patent filed in 2013 and granted in the US in 2021 describes a recessive, non-race-specific resistance gene in pepper called “xcv-1”, which encodes a cysteine-rich transmembrane region with the resistant allele containing a double leucine deletion (Kiss et al., 2021). Interestingly, one of the polymorphic genes located towards the center of the *bs5* fine mapped interval (GeneID: 107864425) encodes a cysteine-rich transmembrane domain-containing protein (CYSTM) and has a double leucine deletion in the resistant allele (**Table 1**). The genomic localization of *xcv-1* has not been reported; however, out of 6 cysteine-rich transmembrane genes annotated in the *C. annuum* UCD10X genome, two are present in the *bs5* region (**Supplementary Table 17**), and only 107864425 is polymorphic between ECW and ECW50R with a double leucine deletion (**Table 1**). Thus, it is likely that *xcv-1* and *bs5* are identical resistances (Szarka et al., 2022) and are encoded by gene 107864425. CYSTM proteins are known to have a role in stress tolerance and disease resistance. Ectopic overexpression of a group of pathogen-induced CYSTM proteins in *Arabidopsis* reduced in-planta population of *Pseudomonas syringae* pv. *tomato* (Pereira Mendes et al., 2021).

A number of *bs6* candidate resistance genes are ZED1-related kinases (ZRKs), which are members of the broad receptor-like kinase/Pelle family of protein kinases (Shiu et al., 2004). ZRKs belong to family RLCK-XII, which includes several pseudokinases that can participate in biotic defense response (Lewis et al., 2013; Wang et al., 2015; Seto et al., 2017). A tomato ZRK, *JIM2* (*RxopJ4*), provides resistance against bacterial spot of tomato by serving as a decoy target for the type III effector, XopJ4, and consequently activates a ZAR1-mediated defense response (Schultink et al., 2019). Surprisingly, *RxopJ4* is one of several ZRKs located in the syntenic region of *bs6* in tomato genome (data not shown) (Sharlach, 2013). Since ZRKs can be targeted by bacterial effectors, and since recessive resistances such as *bs6* often result from modification of bacterial susceptibility targets, four ZRKs in the *bs6* interval are also intriguing candidates for *bs6*.

*bs5* and *bs6* act synergistically and provide resistance against all races of *Xe*. Together with *bs8*, which provides resistance against *Xg*, they enable development of pepper varieties carrying long-lasting recessive resistance to all known BSP pathogens. Pyramiding of resistance genes also increases stability of resistance, both in terms of durability, and against unfavorable conditions. As an example, *bs5* or *bs6*, alone, provides lower levels of resistance at high temperatures (Vallejos et al., 2010). The next steps are to functionally characterize the candidate genes to identify *bs5*/*bs6*. Identification of the resistance genes will facilitate understanding of the mechanism of resistance, which in turn can contribute to the development of novel disease control strategies. Apart from pepper, development of bacterial spot-resistant tomatoes is highly desirable, and identification of the *bs5*/*bs6* genes will be a crucial step for identifying tomato homologs which can be targeted by gene-editing technologies.

## Materials and methods

### Planting materials and growing conditions

For developing populations segregating for resistance, ECW50R and ECW60R were used as resistant parents for *bs5* and *bs6*, respectively. ECW was used as susceptible parent for both populations. For both resistances, ECW was crossed with respective resistant parent to produce an F_1_ population, which was self-pollinated to generate F_2_ seeds. F_3_ populations were generated by selfing of F_2_s when necessary. F_2_ recombinant individuals were self-pollinated, and progeny were genotyped to identify plants fixed for the recombined chromosomal segments (recombinant inbred lines (RILs)). A complete outline of all populations is presented in **Supp. Image 1**. For all plants, seeds were sown in a seedling flat, and fourteen-day-old seedlings were transplanted to 10-cm pots containing Fafard Mix 4 (Fafard, Inc., Agawam, MA). For fine-mapping F_2_ populations, the plants were grown in 242-well trays (Speedling Inc., Sun City, FL) containing Speedling peat-lite soilless media (Speedling Inc., Sun City, FL). The transplants were grown in a greenhouse at temperatures ranging between 20-30 °C.

### Inoculation and disease evaluation

As the resistant responses due to *bs5* and *bs6* do not result in HR induction, they are differentiated from the susceptible response by infiltration of bacterial suspension into pepper leaves at a low concentration (Stall, 1981). In contrast to the development of necrotic lesion in susceptible pepper, the *bs5* resistance only causes a slight yellowing of the infiltrated area and the *bs6* resistant response is characterized by a more intense chlorosis (Vallejos et al., 2010). *Xe* race P6 strain Xv157 was grown in nutrient broth (BBL, Cockeysville, MD) overnight at 28 °C with constant shaking. Bacterial cells were pelleted by centrifugation, the supernatant was discarded, and the cells were re-suspended in sterile tap water. The bacterial suspension was adjusted using Spectronic 20 Genesys spectrophotometer (Spectronic Instruments, Rochester, NY) to OD_600_ = 0.3, which is approximately 10^8^ CFU/ml, then diluted to 10^5^ CFU/ml in sterile tap water. The resulting bacterial suspension was infiltrated with a syringe and hypodermic needle into the mesophyll of the first and second true leaf of five- to six-week-old pepper plants. Inoculated plants were maintained in a greenhouse for disease development, and the plants were evaluated three weeks after inoculation. Plants showing confluent necrosis were rated as susceptible, else they were rated as resistant for the respective resistance. For *bs6* resistance, the disease screen of each RIL was repeated multiple times to obtain accurate phenotypic result.

### GBS library preparation and sequencing

Foliar tissue from young leaves was lyophilized and used for DNA extraction. Genomic DNA was extracted using the Qiagen Plant DNeasy Mini Kit (Qiagen, Germantown, MD) according to the manufacturer’s instructions. The DNA was normalized to 5 ng/µL based on quantification with a Synergy 2 multimode microplate reader (Biotek Instruments, Winooski, VT) with the Quant-iT PicoGreen double-stranded DNA quantification assay (Thermo Fisher Scientific, Waltham, MA). A 96-plex (ninety one F_2_s, a single F_1_, and two each of ECW and respective resistant parent) *ApeKI* GBS library was constructed using a previously published protocol (Elshire et al., 2011). Barcode-adapter titration indicated that 0.9 ng µL^-1^ of each barcode-adapter per 50 ng of genomic DNA produced satisfactory libraries without dimer formation. The barcode-adapter titration mixture and the final GBS library were analyzed on an Agilent 2100 Bioanalyzer (Agilent Technologies, Santa Clara, CA) to ensure acceptable fragment size distribution and quantities. The GBS library was diluted to 3.6 pM and sequenced on one lane (single end, 101 base pair read length) of an Illumina HiSeq 2500 (Illumina Inc, San Diego, CA) at the Genomics Resources Core Facility (Weill Cornell Medicine, NY).

### GBS pipeline and SNP discovery

The raw sequencing reads were processed in TASSEL version 3.0 (Bradbury et al., 2007) using either the reference genome-reliant TASSEL-GBS pipeline (Glaubitz et al., 2014) or the reference-free UNEAK pipeline (for *bs6*) (Lu et al., 2013). For both pipelines, high quality sequencing reads that contained a barcode-adapter, an *ApeKI* restriction site, and an inserted genomic sequence (hereafter termed GBS tags) were identified and selected based on polymorphism between parents. In TASSEL-GBS pipeline, the reads were aligned with the bwa v0.7.8 (Li and Durbin, 2009) to the *C. annuum* UCD10X reference genome, release 1.1 (Hulse-Kemp et al., 2018) to identify polymorphisms (**Supplementary Table 1**). For the UNEAK pipeline, reference genome information was not necessary, and SNPs were identified by pairwise alignment of all unique sequence tags across the entire dataset (**Supplementary Table 2**). Raw read files from sequencing of GBS libraries are deposited in NCBI SRA under bioproject PRJNA863731.

### Linkage analysis

Polymorphic SNPs identified between the parental lines were employed for linkage analyses using MapDisto v1.7 (implemented within Microsoft Excel 2007), (Lorieux, 2012). The parameters in linkage analyses were a minimum LOD=5, a maximum r=0.3, and the ‘Kosambi’ mapping function. The loci were ordered within each linkage map using the auto-order function. QTL analysis was conducted for each population to determine the association between the SNPs within a linkage group and resistance to race P6. Single marker analysis was performed using the R/qtl package in R v3.3.1 (Broman et al., 2003).

### CAPS marker development and genotyping

Cleaved Amplified Polymorphic Sequence (CAPS) markers were designed for validating the mapping results from GBS and for fine mapping. Primers for the markers were designed using Primer 3 software (Untergasser et al., 2007) utilizing SNPs identified from GBS. DNA was extracted using a Cetyltrimethylammonium Bromide (CTAB) method (Doyle and Doyle, 1987) and polymerase chain reaction (PCR) was carried out with Phire Hot Start II DNA polymerase (Thermo Fisher Scientific, Waltham, MA) in a 10 μl volume, which consisted of 2 μl of DNA (adjusted to ~20 ng/μl), 4.89 μl of HPLC-H_2_O, 2 μl of 5X Phire Reaction Buffer, 1 μl of dNTPs, 0.03 μl each of forward and reverse primers, and 0.05 μl of polymerase. The amplicons were digested with appropriate restriction enzymes according to the manufacturer’s recommendations (New England Biolabs, Ipswich, MA). Results were detected using electrophoresis on 3% agarose gels stained with ethidium bromide.

### HRM marker development and genotyping

High Resolution Melting curve (HRM) markers were developed from SNPs identified from GBS. Primers were developed using the IDT PrimerQuest (idtdna.com/Primerquest). DNA was extracted using a NaOH rapid DNA extraction method (Lee et al., 2017). The 5 μl PCR reactions were mixed with 2x AccuStart II PCR SuperMix (Quantabio, Beverly, MA), 0.5 μM of each primer, and 20x EvaGreen Dye (Biotium, Hayward, CA) and run as follows: (95 °C @ 60s) + 40 × ((94 °C @ 5s) + (*Tm* @ 10s) + (72 °C @ 15s)) + (72 °C for 60s), where *Tm* is the annealing temperature. For allele determination, melting curve analysis was performed by scanning the PCR product in a LightCycler 480 Instrument II (Roche, Pleasanton, CA).

### Whole genome sequencing

A modified microprep protocol was used for DNA extraction for whole genome sequencing of ECW60R (Fulton et al., 1995; Sharma et al., 2022b). DNA concentration and purity was verified using NanoDrop (Thermo Fisher Scientific, Waltham, MA). Subsequently, DNA was cleaned using DNeasy PowerClean Pro Cleanup Kit (Qiagen, Germantown, MD) following the manufacturer’s recommendations. Illumina sequencing library was prepared using a Nextera DNA Flex Library Prep Kit (Illumina Inc, San Diego, CA) using the protocol recommended by the manufacturer. The DNA was sequenced to produce 100 base-pairs (bp) paired end reads in one lane of Illumina HiSeq 3000 at University of Florida Interdisciplinary Center for Biotechnology Research.

### Super-scaffolding

The *C. annuum* ECW whole genome sequence (GCA_011745845.1) was only assembled to scaffold level at the time of analysis (Kim et al., 2017). To produce contiguous sequence, the *bs5* or *bs6* fine mapped intervals were blasted against the reference genome *C. annuum* UCD10X (GCF_002878395.1). All ECW scaffolds with query coverage greater than 2% and matching to unique regions were identified and concatenated together in correct order and orientation to produce ECW super-scaffolds for *bs5* and *bs6.* The super-scaffolds also consisted of 5 Kbp region up- and down–stream from flanking markers and 3 Kbp gap between stitched scaffolds. The super-scaffolds were aligned with *C. annuum* UCD10X resistance intervals to verify complete coverage.

### Super-scaffold gene prediction

The ECW genes were predicted *de-novo* to overcome differences in gene annotations between reference genomes. ECW gene prediction model was developed using BRAKER v2.1.6 (Brůna et al., 2021). Within BRAKER, three publicly available ECW RNAseq sequences (SRR13488414, SRR13488423, and SRR13488424) were aligned to *C. annuum* ECW genome sequence (GCA_011745845.1) and supplied to Genemark-ET v4.68 (Lomsadze et al., 2014) to generate hints for training Augustus v3.4.0 (Stanke et al., 2008). The resulting ECW gene prediction model was used to identify potential protein coding regions in the *bs5* and *bs6* super-scaffolds. The genes were validated based on their posterior probability and annotation of homologous regions in *C. annuum* UCD10X or *C. annuum* CM334 annotation.

### Sequence analysis

Polymorphisms for *bs5* were identified using whole genome bulk sequences of PI 163192 × ECW50R F_2_ population, which is fixed for *bs5* gene (Sharma et al., 2022b). For *bs6*, the whole genome sequence of ECW60R was used. The sequences were analyzed using an in-house pipeline. The quality of the reads was verified with FastQC 0.11.7 (bioinformatics.babraham.ac.uk/​projects/​fastqc) and the adapters were trimmed using trim_galore v0.6.5 (Krueger et al., 2021). The trimmed reads were aligned to *C. annuum* ECW genome using Bwa-mem2 v2.2.1 (Vasimuddin et al., 2019). The resulting alignment file was used for variant calling with the HaplotypeCaller tool in GATK 4 (DePristo et al., 2011). The variants were filtered under high stringency as follows: depth ≥ 12, quality-normalized depth ≥ 10, mapping quality ≥ 50, and reference allele depth ≤ 0.1 × alternate allele depth. The sequencing data for PI 163192 × ECW50R F_2_s has previously been deposited in NCBI/​ENA/​DDBJ database under bioproject PRJNA789991. ECW60R whole genome sequence is deposited under bioproject PRJNA863893.

### Candidate genes identification

The coordinates and allelic sequence of high-quality polymorphisms in *bs5*/​*bs6* super-scaffolds were derived from variant calling of *C. annuum* ECW scaffolds with an in-house script. The polymorphism were annotated with snpEff v5.0 (Cingolani et al., 2012) using a custom super-scaffold variant annotation database built using previously described sequences and protein coding regions. Only the variations that result in protein coding changes were selected to identify potential candidate genes. Potential homologs of candidate genes in other *C. annuum* genomes were identified by blasting the predicted amino acid sequences of those genes, which also provided the functional annotations of the candidates. Finally, protein domains containing the polymorphisms between ECW and ECW50R/​ECW60R were identified by Pfam (Mistry et al., 2021) and InterPro search (Blum et al., 2021).

## Data availability statement

The datasets presented in this study can be found in online repositories. The names of the repository/repositories and accession number(s) can be found in the article/**Supplementary Material**.

## Author contributions

GM, JJ, and RS initially developed ECW50R and ECW60R lines. JL, JH, and MM performed GBS and contributed to its analysis. Fine mapping was conducted by JL, RW and SH (genotyping) and GM and JJ (phenotyping). UG generated the whole genome sequences. AS contributed to manuscript writing, fine-mapping, sequence analysis, and identification of candidate genes. All authors contributed to the article and approved the submitted version.
