# Supplementary material for: Mapping of the bs5 and bs6 non-race-specific recessive resistances against bacterial spot of pepper
Source: Front Plant Sci. 2023 May 19;14:1061803. doi: 10.3389/fpls.2023.1061803 (PMC10235544; doi:10.3389/fpls.2023.1061803)
Supplement: Supplementary file 1 [file DataSheet_1.zip › Supplementary alignments.pdf]

Alignment S1. Amino acid alignments of polymorphic genes in *bs5* interval: **g4_CMP-sialic_acid_transporter_2**

```
ECW     MKNGMAECAVCHSRLVSPTVKTISRAYDRHRSKISSKQRALNVLLVVGDC   50
        ||||||||||||||||||||||||||||||||||||||||||||||||||
ECW50R  MKNGMAECAVCHSRLVSPTVKTISRAYDRHRSKISSKQRALNVLLVVGDC   50

ECW     MLVGLQPVLVFMSKVDGKFKFSPVSVNFLTEATKVVFAIIMLLIQARHQK  100
        ||||||||||||||||||||||||||||||||||||||||||||||||||
ECW50R  MLVGLQPVLVFMSKVDGKFKFSPVSVNFLTEATKVVFAIIMLLIQARHQK  100

ECW     VGEKPLLSISTFVQAARNNVLLAVPALLYAINNYLKFIMQLYFNPATVKM  150
        ||||||||||||||||||||||||||||||||||||||||||||||||||
ECW50R  VGEKPLLSISTFVQAARNNVLLAVPALLYAINNYLKFIMQLYFNPATVKM  150

ECW     LSNLKVLVIAMLLKLIMKRRFSVIQWEALALLLIGISINQLRSLPEGATS  200
        ||||||||||+|||||||||||||||||||||||||||||||||||||||
ECW50R  LSNLKVLVIAVLLKLIMKRRFSVIQWEALALLLIGISINQLRSLPEGATS  200

ECW     LALPVTTIAYIYTLVFVTVPSMASVFNEYALKSQYDTSIYLQNLFLYGYG  250
        ||||||||||||||||||||||||||||||||||||||||||||||||||
ECW50R  LALPVTTIAYIYTLVFVTVPSMASVFNEYALKSQYDTSIYLQNLFLYGYG  250

ECW     AIFNFLAILGIAVFKGPGSLDIFQGHSKATMLLIVNNAAQGILSSFFFKY  300
        | ||||||||||||||||||||||||||||||||||||||||||||||||
ECW50R  ATFNFLAILGIAVFKGPGSLDIFQGHSKATMLLIVNNAAQGILSSFFFKY  300

ECW     ADTILKKYSSTVATIFTGIASALLFGHRLTANFLLGISVVFISMHQFFSP  350
        ||||||||||||||||||||||||||||||||||||||||||||||||||
ECW50R  ADTILKKYSSTVATIFTGIASALLFGHRLTANFLLGISVVFISMHQFFSP  350

ECW     LAKVKDDQQNGSLELIDARENHRSKDSSFVNIAAGANEEASHRVGPDERQ  400
        ||||||||||||||||||||||||||||||||||||||||||||||||||
ECW50R  LAKVKDDQQNGSLELIDARENHRSKDSSFVNIAAGANEEASHRVGPDERQ  400

ECW     PLLPR                                               405
        |||||
ECW50R  PLLPR                                               405
```

Alignment S2. Amino acid alignments of polymorphic genes in *bs5* interval:

g16_diacylglycerol_lipase-β

```
ECW     MEGGLQFVWDAIGGFLSSLLGVGCECEGYGVRIVGHSLGGAIAALLGMKL   50
        ||||||||||||||||||||||||||||||||||||||||||||||||||
ECW50R  MEGGLQFVWDAIGGFLSSLLGVGCECEGYGVRIVGHSLGGAIAALLGMKL   50

ECW     RKQYPDLHVYTYGALPCVGLVVADACSEFITSIVNNDEFSARLSVASIMR  100
        ||||||||||||||||||||||||||||||||||||||||||||||||||
ECW50R  RKQYPDLHVYTYGALPCVGLVVADACSEFITSIVNNDEFSARLSVASIMR  100

ECW     LQAAALKALSEDGTIDITTILKLAHHFTSLSVCQKSMSEGESSVNSFTAM  150
        ||||||||||||||||||||||||||||||||||||||||||||||||||
ECW50R  LQAAALKALSEDGTIDITTILKLAHHFTSLSVCQKSMSEGESSVNSFTAM  150

ECW     SSCTNQINHSQLENGLTKREPGSSVLHDIVSSDDKIGSPESSNHFSSLCD  200
        ||||||||||||||||||||||||||||||||||||||||||||||||||
ECW50R  SSCTNQINHSQLENGLTKREPGSSVLHDIVSSDDKIGSPESSNHFSSLCD  200

ECW     CSTSDSSPFDDPLNEFLEAVPSSENKSSLSIPELYLPGLVIHIVPQNGGL  250
        ||||||||||||||||||||||||||||||||||||||||||||||||||
ECW50R  CSTSDSSPFDDPLNEFLEAVPSSENKSSLSIPELYLPGLVIHIVPQNGGL  250

ECW     HKPLWRLWRTWERRSRFRAYVAKREAFKEIIVSPHMFLDHLPWRCQNALE  300
        |||||| |||||||||||||||||||||||||||||||||||||||||||
ECW50R  HKPLWRRWRTWERRSRFRAYVAKREAFKEIIVSPHMFLDHLPWRCQNALE  300

ECW     NILKTGQLKTPEDASEIV                                  318
        ||||||||||||||||||
ECW50R  NILKTGQLKTPEDASEIV                                  318
```

Alignment S3. Amino acid alignments of polymorphic genes in *bs5* interval:
g17_CRIB_domain-containing_protein

```
ECW     MKDRSMDKLFVVFPFSLGCSSESSVPVANTNRSQPPYNTKNSVINQVPTK   50
        ||||||||||||||||||||||||||||||||||||||||||||||||||
ECW50R  MKDRSMDKLFVVFPFSLGCSSESSVPVANTNRSQPPYNTKNSVINQVPTK   50

ECW     RQVGEESSSKVKINGFLIRRRISHGVHTLKRNFKGFYQLFVYKEEEIEEM  100
        ||||||||||||||||||||||||||||||||||||||||||||||||||
ECW50R  RQVGEESSSKVKINGFLIRRRISHGVHTLKRNFKGFYQLFVYKEEEIEEM  100

ECW     ----EIGYPTDVKHVTHIGFDGSNKINNNPMIKSWDNSKSLSFPSISIQQ  146
            ||||||||||||||||||||||||||||||||||||||||||||||
ECW50R  IMEMEIGYPTDVKHVTHIGFDGSNKINNNPMIKSWDNSKSLSFPSISIQQ  150

ECW     FELAMATQASGSSRF                                     161
        |||||||||||||||
ECW50R  FELAMATQASGSSRF                                     165
```

Alignment S4. Amino acid alignments of polymorphic genes in *bs5* interval:

g18_LRR_protein_kinase_MSP1-like

```
ECW     MLLCCTQQPVTTGYLVRDMELLQALRDSLVEKRDFIPSWFDTNTAPCNWT   50
        |||| |||||||||||||||||||||||||+|||||||||||||||||||
ECW50R  MLLCFTQQPVTTGYLVRDMELLQALRDSLVQKRDFIPSWFDTNTAPCNWT   50

ECW     GIKCEGEHVIRIDSPCTMTPLNVPFPGNIGKFRSLKNLNLSRCALTGNIP  100
        ||||||| ||||||||||||||||||||||||||||||||||||||||||
ECW50R  GIKCEGERVIRIDSPCTMTPLNVPFPGNIGKFRSLKNLNLSRCALTGNIP  100

ECW     TDIWSLENLETLDLTDNRLTGELPLTISNLRNLRHLVLDENGFSGSLPST  150
        ||||||||||||||||||||||||||||||||||||||||||||||||||
ECW50R  TDIWSLENLETLDLTDNRLTGELPLTISNLRNLRHLVLDENGFSGSLPST  150

ECW     ICELKDLRELSVHANSFSGNLPDEIGNMEKLQSLDFSSNFFSGSLPSSLG  200
        ||||||||||||||||||||||||||||||||||||||||||||||||||
ECW50R  ICELKDLRELSVHANSFSGNLPDEIGNMEKLQSLDFSSNFFSGSLPSSLG  200

ECW     NLTELLYFDAQQNNLTGSIFPEIGKLSKLRILALSSNMLTGPIPPTISHL  250
        ||||||||||||||||||||||||||||||||||||||||||||||||||
ECW50R  NLTELLYFDAQQNNLTGSIFPEIGKLSKLRILALSSNMLTGPIPPTISHL  250

ECW     KQLEVLDLQNCKFTGIPEEISELISLTYLNLAQNEFDGELPSSFGKLESL  300
        ||||||||||||||||||||||||||||||||||||||||||||||||||
ECW50R  KQLEVLDLQNCKFTGIPEEISELISLTYLNLAQNEFDGELPSSFGKLESL  300

ECW     VYLIASNAGLSGTIPSDLGNCKRLKIINLSFNSFSGALPDELSGLDSLQS  350
        ||||||||||||||||+|||||||||||||||||||||||||||||||||
ECW50R  VYLIASNAGLSGTIPSELGNCKRLKIINLSFNSFSGALPDELSGLDSLQS  350

ECW     LVLDSNRLSGPLPIWISNWTQVESIMVSKNFLSGPLPPLYLPLLSILDVS  400
        ||||||||||||||||||||||||||||||||||||||||||||||||||
ECW50R  LVLDSNRLSGPLPIWISNWTQVESIMVSKNFLSGPLPPLYLPLLSILDVS  400

ECW     ANRLSGELSSGICGAKSLSILLLSDNNFAGDIQNTFRNCSSLTDLVLSGN  450
        ||||||||||||||||||||||||||||||||||||||||||||||||||
ECW50R  ANRLSGELSSGICGAKSLSILLLSDNNFAGDIQNTFRNCSSLTDLVLSGN  450

ECW     NLSGNLPAYLGELQLITLELSKNQFSGKVPDQLWESKSLMGISLSNNMLE  500
        ||||||||||||||||||||||||||||||||||||||||||||||||||
ECW50R  NLSGNLPAYLGELQLITLELSKNQFSGKVPDQLWESKSLMGISLSNNMLE  500

ECW     GPISATIEKLPTLQRIQLDNNQFEGSIPRTIGNLKNLTNLSLHANKLTGG  550
        ||||||||||||||||||||||||||||||||||||||||||||||||||
ECW50R  GPISATIEKLPTLQRIQLDNNQFEGSIPRTIGNLKNLTNLSLHANKLTGG  550
```

```
ECW     IPLELFECTKLVSLDLGANSLSGEILREISKLKLLDNLVLSNNQFSGPIP  600
        ||||||||||||||||||||||||||||||||||||||||||||||||||
ECW50R  IPLELFECTKLVSLDLGANSLSGEILREISKLKLLDNLVLSNNQFSGPIP  600

ECW     EEICSGFQNMPLPDSEFTQHYGMLDLSNNELAGSIPHSIKDCIVVTELLL  650
        ||||||||||||||||||||||||||||||||||||||||||||||||||
ECW50R  EEICSGFQNMPLPDSEFTQHYGMLDLSNNELAGSIPHSIKDCIVVTELLL  650

ECW     QGNKLTGSIPPEISLLGNLTSLDLSFNSLTGPLFPQLFSMTNLQGLILSH  700
        ||||||||||||||||||||||||||||||||||||||||||||||||||
ECW50R  QGNKLTGSIPPEISLLGNLTSLDLSFNSLTGPLFPQLFSMTNLQGLILSH  700

ECW     NQISGSIPDNLDSMMPSLVKLDLSNNRLSGSLPPSAFSVKSLTYLDISMN  750
        ||||||||||||||||||||||||||||||||||||||||||||||||||
ECW50R  NQISGSIPDNLDSMMPSLVKLDLSNNRLSGSLPPSAFSVKSLTYLDISMN  750

ECW     SFSGSLSFNVRTSSSLLVLNASNNQLSGALDDSLSNLTSLSILDLHNNSI  800
        ||||||||||||||||||||||||||||||||||||||||||||||||||
ECW50R  SFSGSLSFNVRTSSSLLVLNASNNQLSGALDDSLSNLTSLSILDLHNNSI  800

ECW     TDNLPPSLSALASLTYLDLSSNSFQKSFPCGICDIEGLVFSNFSGNKFTG  850
        ||||||||||||||||||||||||||||||||||||||||||||||||||
ECW50R  TDNLPPSLSALASLTYLDLSSNSFQKSFPCGICDIEGLVFSNFSGNKFTG  850

ECW     LAPDVCTKSVKCLPSETVLPPRENYASVPVLSHAFVFGIAFCASLLSLVM  900
        ||||||||||||||||||||||||||||||||||||||||||||||||||
ECW50R  LAPDVCTKSVKCLPSETVLPPRENYASVPVLSHAFVFGIAFCASLLSLVM  900

ECW     LIGVLRWRMLRQEAVLLDRGKGKQGKKADPTSTDELLIKKPKEPLSINLA  950
        ||||||||||||||||||||||||||||||||||||||||||||||||||
ECW50R  LIGVLRWRMLRQEAVLLDRGKGKQGKKADPTSTDELLIKKPKEPLSINLA  950

ECW     TFEQSLLRINPTAIFSATENFSKSYIIGDGGFGTVYKAKLPEGRTIAVKR 1000
        ||||||||||||||||||||||||||||||||||||||||||||||||||
ECW50R  TFEQSLLRINPTAIFSATENFSKSYIIGDGGFGTVYKAKLPEGRTIAVKR 1000

ECW     LNGGHMHGDREFFAEMETIGKVKHENLVPLLGYCVFADERFLIYEYMENG 1050
        ||||||||||||||||||||||||||||||||||||||||||||||||||
ECW50R  LNGGHMHGDREFFAEMETIGKVKHENLVPLLGYCVFADERFLIYEYMENG 1050

ECW     SLDFWLRNQVDAVQVLDWPTRFKICLGSARGLSFLHHGFVPHIIHRDIKS 1100
        ||||||||||||||||||||||||||||||||||||||||||||||||||
ECW50R  SLDFWLRNQVDAVQVLDWPTRFKICLGSARGLSFLHHGFVPHIIHRDIKS 1100
```

```
ECW     SNILLDRNFEPRVSDFGLARIISACESHVSTILAGTFGYIPPEYGQTMIA 1150
        ||||||||||||||||||||||||||||||||||||||||||||||||||
ECW50R  SNILLDRNFEPRVSDFGLARIISACESHVSTILAGTFGYIPPEYGQTMIA 1150

ECW     TTKGDIYSFGVVMLELVTGRAPTGQADVEGGNLVGWARWMVANGREIETL 1200
        ||||||||||||||||||||||||||||||||||||||||||||||||||
ECW50R  TTKGDIYSFGVVMLELVTGRAPTGQADVEGGNLVGWARWMVANGREIETL 1200

ECW     DPFFSGSGLWNDQMLRVLAIARLCTNDEPWKRPTMVDVVKLLKEAKNTTN 1250
        ||||||||||||||||||||||||||||||||||||||||||||||||||
ECW50R  DPFFSGSGLWNDQMLRVLAIARLCTNDEPWKRPTMVDVVKLLKEAKNTTN 1250

ECW     T                                                  1251
        |
ECW50R  T                                                  1251
```

Alignment S5. Amino acid alignments of polymorphic genes in *bs5* interval:

g19_ABC_transporter

```
ECW     MGPSGAGKSTLLDGLAGRIASGSLRGKVSMGGLDLSPSFIKRSAAYIMQD   50
        ||||||||||||||||||||||||||||||||||||||||||||||||||
ECW50R  MGPSGAGKSTLLDGLAGRIASGSLRGKVSMGGLDLSPSFIKRSAAYIMQD   50

ECW     DRLFPMLTVYETFMFAADFRLGSLSNTEKQERVERLIEQLGLSSTRNTYI  100
        |||||||||||||||||||||||||||||+||||||||||||||||||||
ECW50R  DRLFPMLTVYETFMFAADFRLGSLSNTEKKERVERLIEQLGLSSTRNTYI  100

ECW     GDEGTRGVSGGERRRVSIGVDIIHGPSLLFLDEPTSGLDSTSAHSVIEKV  150
        ||||||||||||||||||||||||||||||||||||||||||||||||||
ECW50R  GDEGTRGVSGGERRRVSIGVDIIHGPSLLFLDEPTSGLDSTSAHSVIEKV  150

ECW     HDIARAGSTVILTIHQPSHRIQLLLDHLIILARGQLMYQGAPNDVSHHLV  200
        ||||||||||||||||||||||||||||||||||||||||||||||||||
ECW50R  HDIARAGSTVILTIHQPSHRIQLLLDHLIILARGQLMYQGAPNDVSHHLV  200

ECW     RMGRKVPKNESPIEHLIDVIQEYDQSEFGVEAIAAFALTGMKPPPIGAAE  250
        ||||||||||||||||||||||||||||||||||||||||||||||||||
ECW50R  RMGRKVPKNESPIEHLIDVIQEYDQSEFGVEAIAAFALTGMKPPPIGAAE  250

ECW     HEMSSSTVPPSPARTAHRAKYQAVEAHGRDKSSKRLRLQTGAQDDTDFDH  300
        ||||||||||||||||||||||||||||||||||||||||||||||||||
ECW50R  HEMSSSTVPPSPARTAHRAKYQAVEAHGRDKSSKRLRLQTGAQDDTDFDH  300

ECW     SVRSASRSGVLQSIGFSPARRNDHRSSSVMGSSPGCYVYSSEIVQNTPTP  350
        ||||||||||||||||||||||||||||||||||||||||||||||||||
ECW50R  SVRSASRSGVLQSIGFSPARRNDHRSSSVMGSSPGCYVYSSEIVQNTPTP  350

ECW     HSSDYTVNENDYLTPYDFADDTNHSAHDLGPKFANSFFTETWILMRRNFI  400
        ||||||||||||||||||||||||||||||||||||||||||||||||||
ECW50R  HSSDYTVNENDYLTPYDFADDTNHSAHDLGPKFANSFFTETWILMRRNFI  400

ECW     NIMRTPELFLSRLVVLTAMGIMMATMFLRPKENLQGITNRLSFFIFTCTL  450
        ||||||||||||||||||||||||||||||||||||||||||||||||||
ECW50R  NIMRTPELFLSRLVVLTAMGIMMATMFLRPKENLQGITNRLSFFIFTCTL  450

ECW     LFFSSNDAVPAFIQERFVFIRETSHNKYRASSYTIAGLVTYLPFLALQAA  500
        ||||||||||||||||||||||||||||||||||||||||||||||||||
ECW50R  LFFSSNDAVPAFIQERFVFIRETSHNKYRASSYTIAGLVTYLPFLALQAA  500

ECW     VYAVIVWFALSLRGPFIYFLVVLFMSLLSTNSFVIFVSSVVPNYILGYAA  550
        ||||||||||||||||||||||||||||||||||||||||||||||||||
ECW50R  VYAVIVWFALSLRGPFIYFLVVLFMSLLSTNSFVIFVSSVVPNYILGYAA  550
```

```
ECW     VIAFTALFFLFCGYFLDTNDMPSYWKWMNYVSTMTYPYEGLLMNQYQTDE  600
        ||||||||||||||||||||||||||||||||||||||||||||||||||
ECW50R  VIAFTALFFLFCGYFLDTNDMPSYWKWMNYVSTMTYPYEGLLMNQYQTDE  600

ECW     SFGKDPLGRDVTGFGILKSLNISQDSNKKWENLHSSCFLCYIFSLASSFL  650
        ||||||||||||||||||||||||||||||||||||||||||||||||||
ECW50R  SFGKDPLGRDVTGFGILKSLNISQDSNKKWENLHSSCFLCYIFSLASSFL  650

ECW     FRNCTEILKKTAAYSTEKGEFRAGVFELSRFLG                   683
        ||||||||||||||||||||||||||||| |||
ECW50R  FRNCTEILKKTAAYSTEKGEFRAGVFELSWFLG                   683
```

Alignment S6. Amino acid alignments of polymorphic genes in *bs5* interval: **g20_glycine-rich_protein**

```
ECW     MTGKLVVAGEVELEAVAGEDVVTIGEAAGAVATVDMDAVIVVDLVDAVTH   50
        ||||||||||||||||||||||||||||||||||||||||||||||||||
ECW50R  MTGKLVVAGEVELEAVAGEDVVTIGEAAGAVATVDMDAVIVVDLVDAVTH   50

ECW     LLMQKLIIMNIIKSNLEKFYEIHAHVRSYNERVFM                  85
        ||||||||||||||||| | |||||||||||||||
ECW50R  LLMQKLIIMNIIKSNLEMFCEIHAHVRSYNERVFM                  85
```

Alignment S7. Amino acid alignments of polymorphic genes in *bs5* interval:
g23_vacuolar_amino_acid_transporter_1

```
ECW     MCNKSYCKSNGKSLCPHNQVISESNSNKDLESLVYVENQNNVKSNTSFLH   50
        ||||||||||||||||||||||||||||||||||||||||||||||||||
ECW50R  MCNKSYCKSNGKSLCPHNQVISESNSNKDLESLVYVENQNNVKSNTSFLH   50

ECW     AVINMIGMLIGLGQLSTPYALENGGWISSILLIGLGIICAYTSYLLGKCL  100
        ||||||||||||||||||||||||||||||||||||||||||||||||||
ECW50R  AVINMIGMLIGLGQLSTPYALENGGWISSILLIGLGIICAYTSYLLGKCL  100

ECW     QKNPKSKDYKDIGHQAFGTKGRIIVTSFIYFEIFMALISYTISLHDNLSI  150
        ||||||||||||||||||||||||||||||||||||||||||||||||||
ECW50R  QKNPKSKDYKDIGHQAFGTKGRIIVTSFIYFEIFMALISYTISLHDNLSI  150

ECW     VFINTNLTLKGILIHLSTSQILTIIAILVALPSLWLRNFSSISFLSSVGI  200
        ||||||||||||||||||||||||||||||||||||||||||||||||||
ECW50R  VFINTNLTLKGILIHLSTSQILTIIAILVALPSLWLRNFSSISFLSSVGI  200

ECW     FLSFLIFVTVALTAIFGGIKAKHEIPIIHLQNISSISGLYVFSYGGHIVF  250
        ||||||||||||||||||||||||||||||||||||||||||||||||||
ECW50R  FLSFLIFVTVALTAIFGGIKAKHEIPIIHLQNISSISGLYVFSYGGHIVF  250

ECW     PNIYTAMKDASKYTKVSIVSFSLVTTLYVSLAFMGAKLFGPQVSSQITLS  300
        ||||||||| ||||||||||||||||||||||||||||||||||||||||
ECW50R  PNIYTAMKDPSKYTKVSIVSFSLVTTLYVSLAFMGAKLFGPQVSSQITLS  300

ECW     MPHDKIITKIALWATILTPMTKYALEFAPFAIELEENLPSSMKSKVKMMI  350
        ||||||||||||||||||||||||||||||||||||||||||||||||||
ECW50R  MPHDKIITKIALWATILTPMTKYALEFAPFAIELEENLPSSMKSKVKMMI  350

ECW     RGLVGSILLLVILILALCVPYFEHVLSLTGSLVSVGICLIFPCAFYTKIF  400
        ||||||||||||||||||||||||||||||||||||||||||||||||||
ECW50R  RGLVGSILLLVILILALCVPYFEHVLSLTGSLVSVGICLIFPCAFYTKIF  400

ECW     WGEISKKGLVLNVVLIIIGVFLGVVGTVSSSKLLVRSLKRAHD         443
        |||||||||||||||||||||||||||||||||||||||||||
ECW50R  WGEISKKGLVLNVVLIIIGVFLGVVGTVSSSKLLVRSLKRAHD         443
```

Alignment S8. Amino acid alignments of polymorphic genes in *bs5* interval:
g27_ribosome_biogenesis_protein

```
ECW     MPQGDYIELHTKRYGRRHDHFERKRKKEAREVHKRSQIAQKTLGIKGKML
        ||||||||||||||||||||||||||||||||||||||||||||||||||
ECW50R  MPQGDYIELHTKRYGRRHDHFERKRKKEAREVHKRSQIAQKTLGIKGKML   50

ECW     AKKRYAEKAQMKKTELVPAGRFCMFGLVFACELETEPTLLKSDAWLVAKN  100
        ||||||||||||||||||||||||||||||||||||||||||||||||||
ECW50R  AKKRYAEKAQMKKTELVPAGRFCMFGLVFACELETEPTLLKSDAWLVAKN  100

ECW     VMSFMKIFLASPLFLAMLRLLFYTKVPEDGDWPCMKSHQPDVRLMMMCMK  150
        ||||||||||||||||||||||| |||||||| |||||||||||||||||
ECW50R  VMSFMKIFLASPLFLAMLRLLFYIKVPEDGDWTCMKSHQPDVRLMMMCMK  150

ECW     ILSNTIKQKRKEKAGKWEVPLPKVRPVAEDEMFRVIRSGKRKTKQWKRMV  200
        ||||||||||||||||||||||||||||||||||||||||||||||||||
ECW50R  ILSNTIKQKRKEKAGKWEVPLPKVRPVAEDEMFRVIRSGKRKTKQWKRMV  200

ECW     TKATFVGQGFTRKPPKYERFIRPSGLRFTKAHVTHPELKCTFNLEIIGVK  250
        ||||||||||||||||||||||||||||||||||||||||||||||||||
ECW50R  TKATFVGQGFTRKPPKYERFIRPSGLRFTKAHVTHPELKCTFNLEIIGVK  250

ECW     KNPNGPMYTSLGVMTKGTIIEVNVSELGLVTPAGKVVWGKYAQVTNNPEN  300
        ||||||||||||||||||||||||||||||||||||||||||||||||||
ECW50R  KNPNGPMYTSLGVMTKGTIIEVNVSELGLVTPAGKVVWGKYAQVTNNPEN  300

ECW     DGCINAVLLV                                          310
        ||||||||||
ECW50R  DGCINAVLLV                                          310
```

Alignment S9. Amino acid alignments of polymorphic genes in *bs5* interval: **g32_WD repeat-containing**

```
ECW     MPLERVDQLDYMADDREGTDSSDEFEGGLYNEETNLDEYDMPTKVTDTSA
        ||||||||||||||||||||||||||||||||||||||||||||||||||
ECW50R  MPLERVDQLDYMADDREGTDSSDEFEGGLYNEETNLDEYDMPTKVTDTSA   50

ECW     AQARKGKDIQGIAWEELNVTRQSYRLTRLEQYRNYENIPLSGEAVDKECK  100
        ||||||||||||||||||||||||||||||||||||||||||||||||||
ECW50R  AQARKGKDIQGIAWEELNVTRQSYRLTRLEQYRNYENIPLSGEAVDKECK  100

ECW     QVEKGGNYYEFFYNARSVKPTILHFQLRNLVWATSKHDVYLISNDSLMHW  150
        ||||||||||||||||||||||||||||||||||||||||||||||||||
ECW50R  QVEKGGNYYEFFYNARSVKPTILHFQLRNLVWATSKHDVYLISNDSLMHW  150

ECW     SSISRNLSEVVNFTGRIVPTEKYAGSLLEGLTLTQISTMAVKNRFVVAGG  200
        ||||||||||||||||||||||||||||||||||||||||||||||||||
ECW50R  SSISRNLSEVVNFTGRIVPTEKYAGSLLEGLTLTQISTMAVKNRFVVAGG  200

ECW     FQGELICKNLDKPGVSFCARTTYEDNAITNAIEIYESVSYGPRFMAANND  250
        ||||||||||||||||||||||||||||||||||||||||||||||||||
ECW50R  FQGELICKNLDKPGVSFCARTTYEDNAITNAIEIYESVSYGPRFMAANND  250

ECW     CGVRVYDMERFQQMNHFRFPWPVNVVEKFQTGNCDYHDHQRQHRHLLGHT  300
        ||||||||||||||||||||||||||||||||||||||||||||||||||
ECW50R  CGVRVYDMERFQQMNHFRFPWPVNVVEKFQTGNCDYHDHQRQNRHLLGHT  300

ECW     SMSPDCKLFTVVGDDLHGLLVDSRSGKTVASIIGHLDYSFACAWHPDGHT  350
        ||||||||||||||||||||||||||||||||||||||||||||||||||
ECW50R  SMSPDCKLFTVVGDDLHGLLVDSRSGKTVASIIGHLDYSFACAWHPDGHT  350

ECW     FATGNQDKTCRIWDLRNLSSSTAILKGNIGAARSIRFSSDGRFLVVAEPA  400
        ||||||||||||||||||||||||||||||||||||||||||||||||||
ECW50R  FATGNQDKTCRIWDLRNLSSSTAILKGNIGAARSIRFSSDGRFLVVAEPA  400

ECW     DFVHIYSTESDYKKRQEIDLFGEISGVSLSPDDESLYIGIWDRTYGSLLE  450
        ||||||||||||||||||||||||||||||||||||||||||||||||||
ECW50R  DFVHIYSTESDYKKRQEIDLFGEISGVSLSPDDESLYIGIWDRTYGSLLE  450

ECW     YNRRRACRNTKVVDKENEILKDS-SMFLFSDLHIKAGPRGSGLVEFVRLD  499
        ||||||||||||||||||||||  ++|+|
ECW50R  YNRRRACRNTKVVDKENEILKDKYNVFVF                       479

ECW     LV                                                  501

ECW50R                                                      479
```

Alignment S10. Amino acid alignments of polymorphic genes in *bs5* interval:
g33_cysteine-rich_transmembrane_domain_protein

```
ECW     MSYYNQQQPPVGVPPPQGYPPEGYPKDSYPPPGYPQQGYPQQGYPPQGYP   50
        ||||||||||||||||||||||||||||||||||||||||||||||||||
ECW50R  MSYYNQQQPPVGVPPPQGYPPEGYPKDSYPPPGYPQQGYPQQGYPPQGYP   50

ECW     PQYAPQYGAPPPQQQHQSSSSTGLLQGCLAALCCCCLLDACF           92
        ||||||||||||||||||||||||||||||||||||  ||||
ECW50R  PQYAPQYGAPPPQQQHQSSSSTGLLQGCLAALCCCC--DACF           90
```

Alignment S11. Amino acid alignments of polymorphic genes in *bs5* interval: **g44_ATP-dependent_DNA_helicase_2_subunit_KU70**

```
ECW     MSMELDPDEVFGDEEDDPETEFFKEREATKELLVYLVDASPKMFSTTCPS   50
        ||||||||||||||||||||||||||||||||||||||||||||||||||
ECW50R  MSMELDPDEVFGDEEDDPETEFFKEREATKELLVYLVDASPKMFSTTCPS   50

ECW     DDEKTATHFQVAINSIAQSLKSQIINRSYDEVSICFFNTREKKNLQDLSG  100
        ||||||||||||||||||||||||||||||||||||||||||||||||||
ECW50R  DDEKTATHFQVAINSIAQSLKSQIINRSYDEVSICFFNTREKKNLQDLSG  100

ECW     IYVFNVPEREDLDRPTARLIKEFDLIEDLPKGTRLSNICRRTHIHRLFTV  150
        ||||||||||||||||||||||||||||||||||||||||||||||||||
ECW50R  IYVFNVPEREDLDRPTARLIKEFDLIEDLPKGTRLSNICRRTHIHRLFTV  150

ECW     AVRERFAKDIGSKYGIVPGSRENSLYNALWVAQALLRKGSAKTADKRILL  200
        ||||||||||||||||||||||||||||||||||||||||||||||||||
ECW50R  AVRERFAKDIGSKYGIVPGSRENSLYNALWVAQALLRKGSAKTADKRILL  200

ECW     FTNEDDPFGNLKGVVKVDMMRTTLQRAKDAQDLGIAIELLPLSQPDDEFN  250
        ||||||||||||||||||||||||||||||||||||||||||||||||||
ECW50R  FTNEDDPFGNLKGVVKVDMMRTTLQRAKDAQDLGIAIELLPLSQPDDEFN  250

ECW     VSLFYADLLGLEGDDLAQFKALVGERFEDLKDQLRKRIFKKRRVRRLRLV  300
        ||||||||||||||||||||||||||||||||||||||||||||||||||
ECW50R  VSLFYADLLGLEGDDLAQFKALVGERFEDLKDQLRKRIFKKRRVRRLRLV  300

ECW     IFNGLSIELNTYALIRPTNPGRITWLDSMTNLPLKIERSFICADTAAIIQ  350
        ||||||||||||||||||||||||||||||||||||||||||||||||||
ECW50R  IFNGLSIELNTYALIRPTNPGRITWLDSMTNLPLKIERSFICADTAAIIQ  350

ECW     EPPKRFQSYKNENIMFSVAELSEVKRVSTGHLRLLGFKPLTCLKDYHNLK  400
        ||||||||||||||||||||||||||||||||||||||||||||||||||
ECW50R  EPPKRFQSYKNENIMFSVAELSEVKRVSTGHLRLLGFKPLTCLKDYHNLK  400

ECW     PATFVFPSDEEVIGGTCLFVALHRSMVRLKRFAVAFYGSSSHPQLVALVA  450
        ||||||||||||||||||||||||||||||||||||||||||||||||||
ECW50R  PATFVFPSDEEVIGGTCLFVALHRSMVRLKRFAVAFYGSSSHPQLVALVA  450

ECW     QLHTDPNSVPHATDDQIKKASALVKRIDLKDFSVCQFANPALQRHYAVLQ  500
        ||||||||||||||||||||||||||||||||||||||||||||||||||
ECW50R  QLHTDPNSVPHATDDQIKKASALVKRIDLKDFSVCQFANPALQRHYAVLQ  500

ECW     ALALDEDEMPEIKDETLPDEEGMARPGIVKALEEFKLSVYGENYEEEDSN  550
        ||||||||||||||||||||||||||||||||||||||||||||||||||
ECW50R  ALALDEDEMPEIKDETLPDEEGMARPGIVKALEEFKLSVYGENYEEEDSN  550
```

```
ECW     IEGKVEPTRKRKANAMKEYGNYDWADLADNGKLKDLTVVELKYYLGAHNL  600
        ||||||||||||||||||||||||||||||||||||||||||||||||||
ECW50R  IEGKVEPTRKRKANAMKEYGNYDWADLADNGKLKDLTVVELKYYLGAHNL  600

ECW     PVTGKKEVLISRILTHMGNKNDYSLFTKCDDGSLTVLAVYVDDILLAGDS  650
        ||||||||||||||||||||||||||||||||||||| ||||||||||||
ECW50R  PVTGKKEVLISRILTHMGNKNDYSLFTKCDDGSLTVLTVYVDDILLAGDS  650

ECW     VAELDSLKLFLDDQFKIKDLGLLLISRCWLTLILTGLLVLSDSDWAACAL  700
        |||||||||||||||||||| |||||||||||||||||||
ECW50R  VAELDSLKLFLDDQFKIKDLSLLLISRCWLTLILTGLLVLFLDDQ       695

ECW     SRRSMTVFFVTLSGSPISWKSKKQPTVSLSSVEAAYRALGKVVAEVSWLI  750

ECW50R                                                      695

ECW     HLLADFGVCISRPIPVYCESHASLHITRNSVFYERTKHIEIDCHYVRDCV  800

ECW50R                                                      695

ECW     NAGLCLPASCLFC                                       813

ECW50R                                                      695
```

Alignment S12. Amino acid alignments of polymorphic genes in *bs5* interval:
g46_putative_late_blight_resistance_protein_R1B-16

```
ECW     MVNVLTGKSTPAGATASTYGSFLSVDEEIVGFQDDVESIIQQLTKGTKEL   50
        |||||||||||| |||||||||||||||||||||||||||||||||||||
ECW50R  MVNVLTGKSTPARATASTYGSFLSVDEEIVGFQDDVESIIQQLTKGTKEL   50

ECW     DVVSIVGMPGLGKTTLATMVFNHHVIDKYFDVRASCSISKEYNLRKVCSE  100
        ||||||||||||||||||||||||||||||||||||||||||||||||||
ECW50R  DVVSIVGMPGLGKTTLATMVFNHHVIDKYFDVRASCSISKEYNLRKVCSE  100

ECW     ILKQVLGNVDGILDEDMPDKLRKSLMRKRYLIVLDDIWEVKAWEEFRLCF  150
        ||||||||||||||||||||||||||||||||||||||||||||||||||
ECW50R  ILKQVLGNVDGILDEDMPDKLRKSLMRKRYLIVLDDIWEVKAWEEFRLCF  150

ECW     QDDENGSRIMLTTRDEEVARQIKHHSDPYFLRFLKMDESWKLLQKKVFQG  200
        |||||||||||||||||||||||||||||||||| |||||||||||||||
ECW50R  QDDENGSRIMLTTRDEEVARQIKHHSDPYFLRFLTMDESWKLLQKKVFQG  200

ECW     EICPSELRGAGLRVAKSCKGLPLVIVLIAGIIAKQMRASSWLEIAKDLSS  250
        |||||||||||||||||||||||||||||||||||+||||||||||||||
ECW50R  EICPSELRGAGLRVAKSCKGLPLVIVLIAGIIAKQVRASSWLEIAKDLSS  250

ECW     HVLEEQSTKIIESSYNYLEDHLKSCLLYMGLFPEDYKFPVSNLLKLWIAE  300
        ||||||||||||||||||||||||||||||||||||||||||||||||||
ECW50R  HVLEEQSTKIIESSYNYLEDHLKSCLLYMGLFPEDYKFPVSNLLKLWIAE  300

ECW     DFVHDIMVLDNMDMEEASKTCLNDLVNRSLVIVSERREDNGEIEYCTVHD  350
        ||||||||||||||||||||||||||||||||||||||||||||||||||
ECW50R  DFVHDIMVLDNMDMEEASKTCLNDLVNRSLVIVSERREDNGEIEYCTVHD  350

ECW     VVREFCLRKLPKEKFMQYHHAKKHRLSDDDFNEEFLYRYEDALKKLTLED  400
        ||||||||||||||||||||||||||||||||||||||||||||||||||
ECW50R  VVREFCLRKLPKEKFMQYHHAKKHRLSDDDFNEEFLYRYEDALKKLTLED  400

ECW     VDDNLDEQFLQQDEDLLDKSMLKYAHDDLDDDLAEQFLRQDEDLLDKSML  450
        ||||||||||||||||||||||||||||||||||||||||||||||||||
ECW50R  VDDNLDEQFLQQDEDLLDKSMLKYAHDDLDDDLAEQFLRQDEDLLDKSML  450

ECW     EHVHDDLDEDLAEQFSHQDEDLLDMPMLKYVHDDLDEDLDEQFSQDDEYL  500
        ||||||||||||||||||||||||||||||||||||||||||||||||||
ECW50R  EHVHDDLDEDLAEQFSHQDEDLLDMPMLKYVHDDLDEDLDEQFSQDDEYL  500

ECW     WDNPKYVHVNLDEQFLPYEDLSDKRKYVHDDLGKRLLRYQQQGESVEQDE  550
        ||||||||||||||||||||||||||||||||||||||||||||||||||
ECW50R  WDNPKYVHVNLDEQFLPYEDLSDKRKYVHDDLGKRLLRYQQQGESVEQDE  550
```

```
ECW     LIDDPEFSPGDEISLFHVLDNLKFIRVLHLLDVDLKRHSWATAVQVVTHL  600
        |||||||||||||||||||||||||||||||||||||||||| |||||||
ECW50R  LIDDPEFSPGDEISLFHVLDNLKFIRVLHLLDVDLKRHSWATVVQVVTHL  600

ECW     RYLAICTQEFDFKWVSHLHDLQTLQVVRKDDLLGHLQTSPSFWKMQKLRH  650
        ||||||||||||||||||||||||||||||||||||||||||||||||||
ECW50R  RYLAICTQEFDFKWVSHLHDLQTLQVVRKDDLLGHLQTSPSFWKMQKLRH  650

ECW     VDIQDFSFKWEDNDRAIFEESSETVLPNLKTFGKCRIYLADKTPEFWWRF  700
        ||||||||||||||||||||||||||||||||||||||||||||||||||
ECW50R  VDIQDFSFKWEDNDRAIFEESSETVLPNLKTFGKCRIYLADKTPEFWWRF  700

ECW     PNIEQLKLHFIEPGYEVNMPNLEELPVQSLELCFSRPISGYKSTGRANCV  750
        ||||||||||||||||||||||||||||||||||||||||||||||||||
ECW50R  PNIEQLKLHFIEPGYEVNMPNLEELPVQSLELCFSRPISGYKSTGRANCV  750

ECW     VFPSNLNDLSLDRLCLTEKTVSQLAKLQNLESLKLRDVYFKSEDILQPGY  800
        ||||||||||||||||||| ||||||||||||||||||||||||||||||
ECW50R  VFPSNLNDLSLDRLCLTEKAVSQLAKLQNLESLKLRDVYFKSEDILQPGY  800

ECW     STCWDVSDYEFQALKYLNLQNVLVTEWRSSEASFPVLEQLILNNCVIQCG  850
        ||||||||||||||||||||||||||||||||||||||||||||||||||
ECW50R  STCWDVSDYEFQALKYLNLQNVLVTEWRSSEASFPVLEQLILNNCVIQCG  850

ECW     QIPCNFVDIPTLKLIKLIHCDRSLGFSALNIKKEVEEISGCDSLQVLSQY  900
        ||||||||||||||||||||||||||||||||||||||||||||||||||
ECW50R  QIPCNFVDIPTLKLIKLIHCDRSLGFSALNIKKEVEEISGCDSLQVLSQY  900

ECW     KRYYGRLPFGFNHKRGDELISGQWLREGAALIKFVDEHESKLSSSDGYYY  950
        ||||||||||||||||||||||||||||||||||||||||||||||||||
ECW50R  KRYYGRLPFGFNHKRGDELISGQWLREGAALIKFVDEHESKLSSSDGYYY  950

ECW     AYIPKFAWRYRREGCMFCVASHGKAEKKRHACRLTQDNLQKTDFLECIPE 1000
        ||||||||||||||||||||||||||||||||||||||||||||||||||
ECW50R  AYIPKFAWRYRREGCMFCVASHGKAEKKRHACRLTQDNLQKTDFLECIPE 1000

ECW     PSPLVLALPCCLTTAEPVFEYAGRIRMDNVRV                   1032
        ||||||||||||||||||||||||||||||||
ECW50R  PSPLVLALPCCLTTAEPVFEYAGRIRMDNVRV                   1032
```

Alignment S13. Amino acid alignments of polymorphic genes in *bs5* interval:
g50_protein_of_unknown_function

```
ECW     MNTRRRRKICLVEAVEKDSEFEIDADKAREALRKLDEQLQSLSQKQIDPP   50
        |||||||||||||||||||||||||||||||||||||||||||||||||
ECW50R  MNTRRRRKICLVEAVEKDSEFEIDADKAREALRKLDEQLQSLSQKQIDPL   50

ECW     KIRATEVTRASREVTESTNDLEGSFLTTLAFGLLLFTIFYNILFSTVIKP  100
        |||||+||||||||||||||||||||||||||||||||||||||||||||
ECW50R  KIRATDVTRASREVTESTNDLEGSFLTTLAFGLLLFTIFYNILFSTVIKP  100

ECW     AIDGPETVAEVDLYASNS                                  118
        ||||||||||||||||||
ECW50R  AIDGPETVAEVDLYASNS                                  118
```

Alignment S14. Amino acid alignments of polymorphic genes in *bs5* interval: **g51_pirin-like_protein**

```
ECW     MSESNEYDSCFDNPRLVIKKVLAKPQSEGNGAVVRRSIGRHELRNLDPFL   50
        ||||||||||||||||||||||||||||||||||||||||||||||||||
ECW50R  MSESNEYDSCFDNPRLVIKKVLAKPQSEGNGAVVRRSIGRHELRNLDPFL   50

ECW     ILDEFSVSAPAGFPDHPHRGFETVTYMLEGAFTHQDFAGHKGTINTGDVQ  100
        +|||||||||||||||||||||||||||||||||||||||||||||||||
ECW50R  VLDEFSVSAPAGFPDHPHRGFETVTYMLEGAFTHQDFAGHKGTINTGDVQ  100

ECW     WMTAGRGIIHSEMPAGEGSQKGLQLWINLSSKDKMIEPRYQELLDEDIPR  150
        ||||||||||||||||||||||||||||||||||||||||||||||||||
ECW50R  WMTAGRGIIHSEMPAGEGSQKGLQLWINLSSKDKMIEPRYQELLDEDIPR  150

ECW     AENNGVKVKIIAGEAMGVQSPVYTRTPTMYLDFTLQPTSYHHQAIPESWN  200
        ||||||||||||||||||||||||||||||||||||||||||||||||||
ECW50R  AENNGVKVKIIAGEAMGVQSPVYTRTPTMYLDFTLQPTSYHHQAIPESWN  200

ECW     AFVYIIEGEGVFGIPNSGPVSAHHCLVLGPGEGLSVWNKSSKPLRFVLLG  250
        ||||||||||||||||||||||||||||||||||||||||||||||||||
ECW50R  AFVYIIEGEGVFGIPNSGPVSAHHCLVLGPGEGLSVWNKSSKPLRFVLLG  250

ECW     GQPLNEPVVQHGPFVMNSQDEIDQTFEDYQYCKNGFENARYWRSGY      296
        ||||||||||||||||||||||||||||||||||||||||||||||
ECW50R  GQPLNEPVVQHGPFVMNSQDEIDQTFEDYQYCKNGFENARYWRSGY      296
```

Alignment S15. Amino acid alignments of polymorphic genes in *bs6* interval:
Formyltetrahydrofolate deformylase

```
ECW     MNLLWRLVTPNIGPQVCKFAKRSLKSVKFPFGSSNSLLHGIHVFHCPDDV   50
        ||||||||||||||||||||||||||||||||||||||||||||||||||
ECW60R  MNLLWRLVTPNIGPQVCKFAKRSLKSVKFPFGSSNSLLHGIHVFHCPDDV   50

ECW     GIVAKLSDCIASKGGNILTADVFVPQDNNVFYSRSEFTFDPAIWPRGEMD  100
        ||||||||||||||||||||||||||||||||||||||||||||||||||
ECW60R  GIVAKLSDCIASKGGNILTADVFVPQDNNVFYSRSEFTFDPAIWPRGEMD  100

ECW     EDFSKLSRMFNAMKSVVRVPEIDPKYKIAVLVSKQDHCLVDLLHRWQNGR  150
        ||||||||||||||||||||||||||||||||||||||||||||||
ECW60R  EDFSKLSRMFNAMKSVVRVPEIDPKYKIAVLVSKQDHCLVDLLHRW      146

ECW     LPVDITSVISNHDRGPDAHVIWLLERHGIPYYYLPTTTGKKREVEILELV  200

ECW60R                                                      146

ECW     HDTDFLVLARYMQVFSAEFLKSYGKDIINIHHGLLPSFKGGSPSKQAFEA  250

ECW60R                                                      146

ECW     GVKLIGATSHFVSEVLDAGAIIEQMVERVSHRDNLRSFTQKSEDLEKQCL  300

ECW60R                                                      146

ECW     VKAIKSYCELRVLPYEKDKTVVF                             323

ECW60R                                                      146
```

Alignment S16. Amino acid alignments of polymorphic genes in *bs6* interval:
Phosphatidylserine decarboxylase proenzyme 1

```
ECW     MKFRAYRGIPFFTYNERFNQRFHFTTLFRKVQATEARASLNPGGSSSNST   50
        ||||||||||||||||||||||||||||||||||||||||||||||||||
ECW60R  MKFRAYRGIPFFTYNERFNQRFHFTTLFRKVQATEARASLNPGGSSSNST   50

ECW     SQGSTLLLPGATVATIVMLGLLHARRLYDDQKIEDAREKGTLEFQPDVKA  100
        |||||||||||||||||||| |||||||||||||||||||||||||||||
ECW60R  SQGSTLLLPGATVATIVMLGFLHARRLYDDQKIEDAREKGTLEFQPDVKA  100

ECW     TFMRLLPLRSVSRFWGTLTSVELPMWLRPSVYKGWARAFHSNLEEVALPL  150
        ||||||||||||||||||||||||||||||||||||||||||||||||||
ECW60R  TFMRLLPLRSVSRFWGTLTSVELPMWLRPSVYKGWARAFHSNLEEVALPL  150

ECW     EEYASLREFFVRRLKEGTRPIDSDPCSLISPVDGTVLQFGELKEAGAMIE  200
        ||||||||||||||||||||||||||||||||||||||||||||||||||
ECW60R  EEYASLREFFVRRLKEGTRPIDSDPCSLISPVDGTVLQFGELKEAGAMIE  200

ECW     QVKGFSYSVSSLLGTSSLLPMTLVDDSTNQDGGKEGSMDDANQKSWWRVS  250
        ||||||||||||||||||||||||||||||||||||||||||||||||||
ECW60R  QVKGFSYSVSSLLGTSSLLPMTLVDDSTNQDGGKEGSMDDANQKSWWRVS  250

ECW     LSSPKVRDPAPARPMKGLFYCVIYLRLGDYHRIHSPADWNVLGRRHFSGR  300
        ||||||||||||||||||||||||||||||||||||||||||||||||||
ECW60R  LSSPKVRDPAPARPMKGLFYCVIYLRLGDYHRIHSPADWNVLGRRHFSGR  300

ECW     LFPVNERATRTIRNLYVENERVVLEGKWQEGYMAMAAVGATNIGSIELFI  350
        ||||||||||||||||||||||||||||||||||||||||||||||||||
ECW60R  LFPVNERATRTIRNLYVENERVVLEGKWQEGYMAMAAVGATNIGSIELFI  350

ECW     EPTLRTNRPWKKLLHPEPPEEQVYEPKGTGVLLKKGDELAAFNMGSTVVM  400
        ||||||||||||||||||||||||||||||||||||||||||||||||||
ECW60R  EPTLRTNRPWKKLLHPEPPEEQVYEPKGTGVLLKKGDELAAFNMGSTVVM  400

ECW     VFQAPILQPSADKSTSTEFSFCVKKGDRVRVGEALGRWRDP           441
        |||||||||||||||||||||||||||||||||||||||||
ECW60R  VFQAPILQPSADKSTSTEFSFCVKKGDRVRVGEALGRWRDP           441
```

Alignment S17. Amino acid alignments of polymorphic genes in *bs6* interval: **ZED1-related kinase (ZRK) 4**

```
ECW60R* MHHLMSALLTTKKLFSSASVKKRKKEQDCYLNNGSAVLEEILALCNGNCR   50
        ||||||||||||||||||||||||||||||||||||||||||||||||||
ECW     MHHLMSALLTTKKLFSSASVKKRKKEQDCYLNNGSAVLEEILALCNGNCR   50

ECW60R                                                        0

ECW60R* IPIRYFTATEIERAIKHSEKKIELDEGYMVTGSLENHLFLIRFSLYSYGV  100
        ||||||||||||||||||||||||||||||||||+|||||||||||||||
ECW     IPIRYFTATEIERAIKHSEKKIELDEGYMVTGSLDNHLFLIRFSLYSYGV  100

ECW60R                                                MAC     3

ECW60R* HRDISNYSSDESSQECAETYWLLSRI                          126
        ||||
ECW     HRDIAITAQMSHLKNVLRLIGCCLEFEEPVMVYEYIEGISLGDLLFEEGN  150
            ||||||||||||||||||||||||||||||||||||||||||||||
ECW60R  TEILAITAQMSHLKNVLRLIGCCLEFEEPVMVYEYIEGISLGDLLFEEGN   53

ECW60R                                                      126

ECW     HDDQTRNSLLSWGSRLRIANEVASALVFLHTEFTTPIIHRDIKPHKVIID  200
        |||||| |||||||||||||||||||||||||||||||||||||||||||
ECW60R  HDDQTRKSLLSWGSRLRIANEVASALVFLHTEFTTPIIHRDIKPHKVIID  103

ECW60R                                                      126

ECW     QNSSIAKIVDFSFSISLPSGELEVQDRVCGTIEYLDPEYGYCGIITQKTD  250
        ||||||||||||||||||||||||||||||||||||||||||||||||||
ECW60R  QNSSIAKIVDFSFSISLPSGELEVQDRVCGTIEYLDPEYGYCGIITQKTD  153

ECW60R                                                      126

ECW     VYSFGVLLFELLTGKELRDIMESNVEEGNVMDRVDSAISEETINFENFLP  300
        ||||||||||||||||||||||||||||||||||||||||||||||||||
ECW60R  VYSFGVLLFELLTGKELRDIMESNVEEGNVMDRVDSAISEETINFENFLP  203

ECW60R                                                      126

ECW     NLIDRYIKEGNIMDIADNAILEEHGIEIQQQLQDYLNLVKKCTTYNRKNR  350
        ||||||||||||||||||||||||||||||||||||||||||||||||||
ECW60R  NLIDRYIKEGNIMDIADNAILEEHGIEIQQQLQDYLNLVKKCTTYNRKNR  253
```

```
ECW60R                                                      126

ECW     PYMIQVAKELCRIHKCFHAFTLGQI                           375
        |||||||||||||||||||||||||
ECW60R  PYMIQVAKELCRIHKCFHAFTLGQI                           278
```

Alignment S18. Amino acid alignments of polymorphic genes in *bs6* interval: **ZRK1-like serine/ threonine-protein kinase**

```
ECW60R  MQVFRELARKKQSSSSASAETLKKEHDYYLENGSAVLRGASCFMRWKLPN   50
        |||||||||||||||||||||||||||||||||||||
ECW     MQVFRELARKKQSSSSASAETLKKEHDYYLENGSAVLEELLALCDGNCRI   50

ECW60R                                                        0

ECW60R  SHPLLHCH                                             58

ECW     PIRYSTATEIEKAIKHPENKMELSYGLRIAHQVVSAVVFLHTEFTTPVIH  100
                            ||||||||||||||||||||||||||||||
ECW60R                      MELSYGLRIAHQVVSAVVFLHTEFTTPVIH   30

ECW60R                                                       58

ECW     RDLKPSNVIVDQNSGVAKLLNFSLSVSLPRGELEVVKDVMCGTYGYLASE  150
        ||||||||||||||||||||||||||||||||||||||||||||||||||
ECW60R  RDLKPSNVIVDQNSGVAKLLNFSLSVSLPRGELEVVKDVMCGTYGYLASE   80

ECW60R                                                       58

ECW     YASSCIVTQKIDVYSFGVVLLQLLTGWKMSTLHLKDRNMDYIVDPSDSDF  200
        ||+|||||||||||||||||||||||||||||||||||||||||||||||
ECW60R  YAASCIVTQKIDVYSFGVVLLQLLTGWKMSTLHLKDRNMDYIVDPSDSDF  130

ECW60R                                                       58

ECW     KMMNIEEIHVMDIADPAILEEHGIAIQQQLKDCWDLVNKCTKSSGKDRPY  250
        ||||||||||||||||||||||||||||||||||||||||||||||||||
ECW60R  KMMNIEEIHVMDIADPAILEEHGIAIQQQLKDCWDLVNKCTKSSGKDRPY  180

ECW60R                                                       58

ECW     MIEVARELHRIHKCFRACP                                 269
        |||||||||||||||||||
ECW60R  MIEVARELHRIHKCFRACP                                 199
```

Alignment S19. Amino acid alignments of polymorphic genes in *bs6* interval: **TCP-1/Cpn-60 chaperonin-like**

```
ECW     MALEFDEFGRSFLIIKEQVQKTRLRGLDAQKANIFAGKVVACILCTSFGL   50
        |||||||||||||||||||||||||||||||||||||||||||||| |||
ECW60R  MALEFDEFGRSFLIIKEQVQKTRLRGLDAQKANIFAGKVVACILCTFFGL   50

ECW     KGMDKMFQSPNGNVTIINNSAHILEQMDVDNQIAKLIIEWSVSQDYEIGD  100
        |||||+||||||||||||||||||||||||||||||||||||||||||||
ECW60R  KGMDKIFQSPNGNVTIINNSAHILEQMDVDNQIAKLIIEWSVSQDYEIGD  100

ECW     GTTEVVVIAGTLLEQAKKLLEHGINPIELQKDMK                 134
        ||||||||||||||||||||||||||||||||||
ECW60R  GTTEVVVIAGTLLEQAKKLLEHGINPIELQKDMK                 134
```

Alignment S20. Amino acid alignments of polymorphic genes in *bs6* interval: **ZRK1-like serine/ threonine-protein kinase**

```
ECW     MHHLMTTPLTMKSLFHPASVKRRKKEQDCYLKNGSAVLEEILALCDGNCR   50
        ||||||||||||||||||||||||||||||||||||||||||||||||||
ECW60R  MHHLMTTPLTMKSLFHPASVKRRKKEQDCYLKNGSAVLEEILALCDGNCR   50

ECW     IPICHFTATEIQRAIKHSEKKIELDEGYMVTGSLDNRLILVRFCSQFYSV  100
        ||||||||||||||||||||||||||||||||||||||||||||||||||
ECW60R  IPICHFTATEIQRAIKHSEKKIELDEGYMVTGSLDNRLILVRFCSQFYSV  100

ECW     HRDIAVTAQTSHLKNVLRLVGCCLEFEEPVMVYEYVKGVALSDLLFGEVV  150
        ||||||||||||||||||||||||||||||||||||||||||||||||||
ECW60R  HRDIAVTAQTSHLKNVLRLVGCCLEFEEPVMVYEYVKGVALSDLLFGEVV  150

ECW     FLHTEFTTPIIHRDIQPHEVIIDQNSSVAKIVDFSLSISLPPGELELQDQ  200
        ||||||||||||||||||||||||||||||||||||||||||||||||||
ECW60R  FLHTEFTTPIIHRDIQPHEVIIDQNSSVAKIVDFSLSISLPPGELELQDQ  200

ECW     PIWGTFWYADPEYAALGIVTQKFDVYSFGVVLFQLLTGKKVSNSMYSNIE  250
        ||||||||||||||||||||||||||||||||||||||||||||||||||
ECW60R  PIWGTFWYADPEYAALGIVTQKFDVYSFGVVLFQLLTGKKVSNSMYSNIE  250

ECW     EGNAVDRGDHEIPVETIDFKEGVEPNTEEGNAMGRVNSAISEEAIYSETK  300
        |||||||||| |||||||||||||||||||||||||||||||||||||||
ECW60R  EGNAVDRGDHVIPVETIDFKEGVEPNTEEGNAMGRVNSAISEEAIYSETK  300

ECW     GLNPVRYFIEKNNVMDIADPTLLEEHGISIQQ                    332
        ||| ||||||||||||||| ||||||||||||
ECW60R  GLNAVRYFIEKNNVMDIADTTLLEEHGISIQQ                    332
```

Alignment S21. Amino acid alignments of polymorphic genes in *bs6* interval: **Ubiquitin conjugating enzyme variant (UEV) 1C-like**

```
ECW     MTLGSGGGSNFVVPQNFRLLKELERGEKGIGDSTVSYGMDGGDDIYMRSW   50
        |||||||||||||||||||||||||||||||||||||||| |||+|||||
ECW60R  MTLGSGGGSNFVVPQNFRLLKELERGEKGIGDSTVSYGMDDGDDMYMRSW   50

ECW     TDTIIGPHNYVHEGRIYQLKLFCDKD                           76
        + ||||||||||||||||||||||||
ECW60R  SGTIIGPHNYVHEGRIYQLKLFCDKD                           76
```

Alignment S22. Amino acid alignments of polymorphic genes in *bs6* interval: **ZED1-related kinase (ZRK) 1**

```
ECW     MQFIKGLTRKKQSSSSASAETRKKEHDYYLENGSAVLEELLALCDGNCRI   50
        ||||||||||||||||||||||||||||||||||||||||||||||||||
ECW60R  MQFIKGLTRKKQSSSSASAETRKKEHDYYLENGSAVLEELLALCDGNCRI   50

ECW     PIRYFTATDIERAIKHSENKLDIADGHMVAGSLDKRLVFIRTFPGYFRNH  100
        ||||||||||||||||||||||||||||||||||||||||||||||||||
ECW60R  PIRYFTATDIERAIKHSENKLDIADGHMVAGSLDKRLVFIRTFPGYFRNH  100

ECW     FNNIFRDIAITAQMSHLKNVLRLVGCCIEFEEPVMVYEYVEATSLYALLF  150
        || |||||||||||||||||||||||||||||||||||||||||||||||
ECW60R  FN-IFRDIAITAQMSHLKNVLRLVGCCIEFEEPVMVYEYVEATSLYALLF  149

ECW     EKDNHDDETRKSLLSWGSRLRIAHKVASAVVFLHTEFTTPIIHRDLKPSN  200
        ||||||||||||||||||||||||||||||||||||||||||||||||||
ECW60R  EKDNHDDETRKSLLSWGSRLRIAHKVASAVVFLHTEFTTPIIHRDLKPSN  199

ECW     VIVDQNSGVAKLLNFSLSVSLPQGKLEVVKDVVCGTYGYVAPEYSEWCMI  250
        ||||||||||||||||||||||||+|||||||||||||||||||||||||
ECW60R  VIVDQNSGVAKLLNFSLSVSLPQGELEVVKDVVCGTYGYVAPEYSEWCMI  249

ECW     TQKIDVYSFGVILLQLLTRRNMTTLHLKDRDMYYIVDPSDSDFKMLNIEE  300
        ||||||||||||||||||||||||||||||||||||||||||||||||||
ECW60R  TQKIDVYSFGVILLQLLTRRNMTTLHLKDRDMYYIVDPSDSDFKMLNIEE  299

ECW     IHVMDIADPAILEEHGIKIRQQLEDCLDLVKKCTAYKGEERPYMIEVAKE  350
        ||||||||||||||||||||||||||||||||||||||||||||||||||
ECW60R  IHVMDIADPAILEEHGIKIRQQLEDCLDLVKKCTAYKGEERPYMIEVAKE  349

ECW     LSRIHHCFRALTLCRN                                    366
        ||||||||||||||||
ECW60R  LSRIHHCFRALTLCRN                                    365
```
