# Supplementary material for: Mapping of the bs5 and bs6 non-race-specific recessive resistances against bacterial spot of pepper
Source: Front Plant Sci. 2023 May 19;14:1061803. doi: 10.3389/fpls.2023.1061803 (PMC10235544; doi:10.3389/fpls.2023.1061803)
Supplement: Supplementary file 1 [file DataSheet_1.zip › Supplementary figures.pdf]

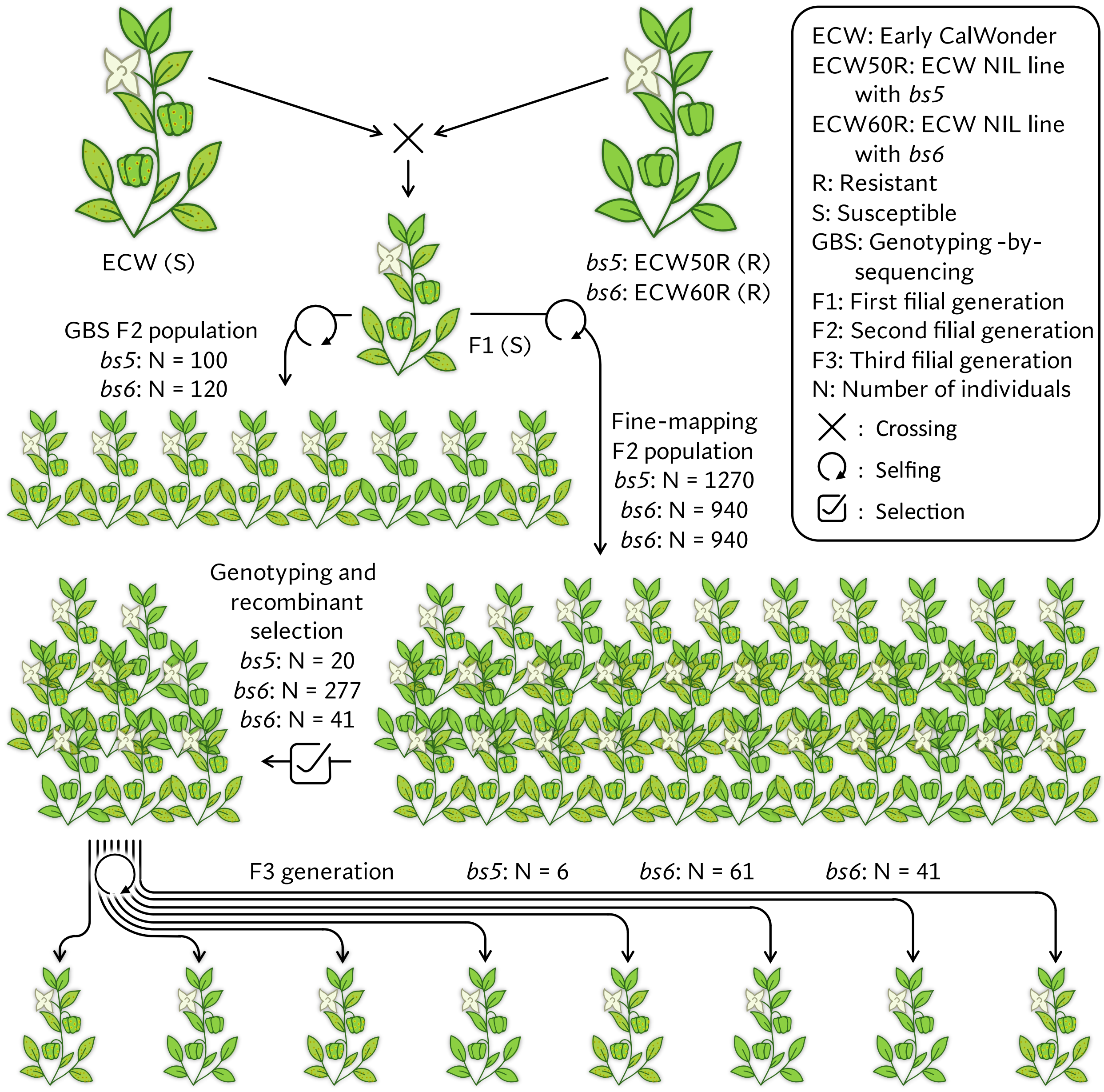

Figure S1. Schematic representations of crosses made to generate GBS and fine-mapping F_2 population for *bs5* and *bs6*.
